# Study Protocol for “Exploring the safety and therapeutic potential of psilocybin in the treatment of anorexia nervosa in adolescents and young adults”

**DOI:** 10.1371/journal.pone.0352246

**Published:** 2026-06-30

**Authors:** David Sjöström, Olea Schau Rybäck, Emma Claesdotter Knutsson, Petri Kajonius, Oskar Jensen Sondén, Per Carlbring, Johannes Björkstrand, Pouya Movahed Rad

**Affiliations:** 1 Department of Clinical Sciences, Faculty of Medicine, Lund University, Lund, Sweden; 2 Adult Psychiatry, Skåne University Hospital, Lund, Sweden; 3 Child and Adolescent Psychiatry, Skåne University Hospital, Lund, Sweden; 4 Department of Psychology, Lund University, Lund, Sweden; 5 Department of Psychology, Stockholm University, Stockholm, Sweden; 6 School of Psychology, Korea University, Seoul, South Korea; Public Library of Science, UNITED STATES OF AMERICA

## Abstract

**Background:**

Anorexia nervosa (AN) is a severe psychiatric disorder with high morbidity, mortality, and relapse rates, most commonly emerging during adolescence. Despite specialized psychological and nutritional treatments, outcomes remain suboptimal, with high rates of relapse and chronicity. Psilocybin has been investigated with preliminary efficacy in other psychiatric conditions characterized by rigidity and treatment resistance, but clinical evidence in AN—particularly in adolescents—is limited.

**Objective:**

The psiAN study aims to evaluate the safety, tolerability, and feasibility of psilocybin therapy combined with psychological support in adolescents and young adults with relapsing AN, while exploring clinical, experiential, and neurobiological correlates of change.

**Methods:**

A phase IIa, open-label, randomized controlled trial enrolling individuals aged 16–35 years with DSM-5 AN and a history of relapse. Participants are randomized to receive either two administrations of psilocybin (25 mg) with manualized psychological support plus treatment as usual (TAU), or TAU alone. Primary outcomes focus on safety and tolerability, assessed through adverse events, psychiatric monitoring, and medical parameters measured from first dosing to primary endpoint. Secondary outcomes include change in eating disorder symptom severity, relapse composite measures, mood, well-being, personality traits from baseline to primary endpoint with follow-up to 12 months. Functional magnetic resonance imaging (fMRI) and peripheral brain-derived neurotrophic factor are included as exploratory mechanistic measures. fMRI will evaluate pre- to post-intervention changes in structural and functional connectivity and task-related responses during a simplified Monetary Incentive Delay task (MIDT) and a Calorie-Cue Task (CCT). ClinicalTrials.gov Identifier: NCT07169747.

**Ethics and dissemination:**

The study follows Good Clinical Practice (GCP), the Declaration of Helsinki, and EU Clinical Trials Regulation requirements, with staged inclusion of adolescents (16–17-year-olds) after a safety board review of adult data (18–35-year-olds). This protocol was prepared with reference to the SPIRIT 2025 guidelines (Chan et al., 2025) to enhance transparency and inform future trials.

## Introduction

### Anorexia nervosa

Anorexia Nervosa (AN) is a severe [1] psychiatric disorder characterized mainly by persistent restriction of food intake, intense fear of weight gain, cognitive rigidity and disturbances in body image [2]. The disorder typically emerges during adolescence or young adulthood with a lifetime prevalence in females up to 4% and in 0.3% in males [3], and is associated with profound psychological suffering, psychiatric and somatic co-morbidity, and the highest mortality rate among psychiatric conditions [4–6]. Despite advances in psychotherapeutic approaches and specialized multidisciplinary care, long-term outcomes remain suboptimal. Approximately one in five individuals with AN develop a severe and enduring course [7], and relapse after remission is common, occurring in roughly 30% within 1–2 years and in 40–50% over even longer follow-up periods [8,9]. Risk of a severe and enduring course in AN is associated with longer duration of untreated illness, persistently low body mass index, psychiatric comorbidity, and cognitive inflexibility, while earlier remission is consistently linked to more favourable long-term outcomes, underscoring the prognostic importance of timely and effective interventions [2,7,8].

Current evidence-based treatments for AN rely primarily on psychological and nutritional interventions, including family-based therapy for adolescents and specialized psychotherapies for adults; however, no pharmacological treatments are approved for core AN psychopathology [10]. These limitations have led to increasing interest in novel interventions targeting transdiagnostic mechanisms implicated in illness persistence, including cognitive inflexibility, maladaptive learning processes related to food and self-image, and emotional avoidance [11].

This need may be particularly salient during adolescence, a developmental period characterized by ongoing cortical maturation and heightened neuroplasticity [12–14]. Neuroimaging studies consistently demonstrate that acute underweight in AN is associated with widespread reductions in cortical thickness and grey matter volume, changes that appear largely related to malnutrition and often show substantial normalization with weight restoration [15]. Importantly, emerging evidence suggests that age and duration of illness influence the extent and trajectory of structural brain recovery, indicating that prolonged underweight during adolescence may intersect with sensitive neurodevelopmental windows [16]. Given that AN most commonly emerges during adolescence and that longer duration of untreated illness is associated with poorer long-term outcomes, early and effective intervention are critical not only for symptomatic recovery but also for preventing the consolidation of maladaptive neural and behavioural patterns. Within this context, careful, staged investigation of innovative interventions for AN in adolescents represents both a clinical and developmental imperative.

### Psilocybin

Classic serotonergic psychedelics such as psilocybin act primarily via agonism at the serotonin 5-HT_2_A receptor [17] and are known to in medium to high doses induce variations of profound, time-limited alterations in perception, cognition, emotion, and self-experience [18]. Over the past decade, controlled clinical trials have demonstrated that psilocybin interventions may produce rapid and sustained improvements in conditions often characterized by high levels of psychological rigidity and treatment resistance, such as depression [19–22], death related anxiety [23–27], and substance use disorders [28,29]. Psilocybin additionally demonstrates a favourable safety profile with low occurrence of serious adverse events in modern clinical trials [30,31].

Beyond symptom reduction, psilocybin experiences are frequently described by patients as personally meaningful and transformative with sometimes enduring changes in perspective, emotional processing, personality, and self-relation [32–34]. Neuropsychological mechanistic models suggest that increased cognitive and emotional flexibility [35] together with the disruption of maladaptive predictive models [36,37] implicated in psychopathology [38], may enhance learning and behavioural updating during a transient period of heightened plasticity [39–41]. These mechanisms of change can hypothetically benefit the recovery in AN, where rigid cognitive styles, compulsive behavioural patterns, and avoidance are central maintaining factors [42].

### Psilocybin and AN

Interest in psychedelic interventions for eating disorders has increased substantially in recent years [23,42–44]. Although the available evidence base remains limited, qualitative and mixed-methods studies (combining self-report measures with in-depth interviews) have reported on therapeutic associations from the naturalistic ceremonial use of ayahuasca, another classical psychedelic, including self-reported reductions in eating-disorder symptomatology associated with increased emotional insight, self-compassion, and enhanced motivation for recovery [45,46]. These preliminary findings align closely with hypothesized mechanisms of change in psychedelic therapy in general, such as increases in cognitive flexibility, enhanced emotional regulation, and mystical experiences with subjective meaning and therapeutic effects. Hypothetically, these self-reported effects might serve as adjunctive or augmentative mechanisms to reduce eating-disorder psychopathology, particularly as adjunctive component with existing biopsychosocial treatment framework [42,47]. Underlying neurobiological mechanisms such as serotonergic modulation [48,49] and plasticity-related signalling pathways, may contribute to both acute and sustained therapeutic effects [50]. Together, the current literature supports the hypothesis that psilocybin therapy may target core mechanisms underlying the persistence of AN rather than merely alleviating secondary symptoms such as comorbid depression or anxiety known to be reduced by psilocybin as well [22,51]. Also, preclinical data lend further support to this rationale. In the activity-based anorexia model, a single dose of psilocybin improved body weight maintenance and enhanced cognitive flexibility in female rats, with effects mediated primarily through 5-HT1A receptor signalling rather than the canonical 5-HT2A pathway alone [52]. These findings suggest that psilocybin may engage disorder-specific mechanisms in AN through multiple serotonergic pathways, and that improvements in flexible behaviour can persist beyond the acute psychedelic experience.

Based on the existing body of evidence, clinical studies have begun to emerge. A phase 1 open-label feasibility study of psilocybin-assisted therapy in adult women with anorexia nervosa found a single 25 mg dose to be safe and well tolerated, with no clinically significant changes in ECG, vital signs, or suicidality, and only transient mild adverse events, including two episodes of asymptomatic low blood glucose [53]. Participants reported subjectively meaningful experiences, and reductions in eating-disorder symptoms were observed at short-term follow-up but not reflected in significant BMI needed for remission. Additionally, most patients wanted a second psilocybin dosing session in the treatment program. Although preliminary, these findings provide early evidence supporting further investigation of psilocybin in this population. A subsequent report from the same trial described the therapeutic emergence of previously dissociated traumatic memories in two of ten participants during psilocybin dosing, both of whom subsequently achieved clinically meaningful improvement in eating disorder symptoms [54]. These observations point to a role for trauma processing in recovery and strengthen the case for trauma-informed preparation and integration support in psilocybin-assisted therapy for AN. Currently, several clinical trials are currently underway exploring the therapeutic potential of psilocybin for patients suffering from AN and other eating disorders (ClinicalTrials.gov Identifier: NCT04052568, NCT06399263, NCT05481736, NCT04505189).

### Functional magnetic resonance imaging and AN

Functional magnetic resonance imaging (fMRI) studies demonstrate that patients with active AN have significantly reduced volumes of both grey and white matter, with more pronounced loss observed in adolescents [55]. These reductions appear to reflect the physiological effects of acute starvation rather than enduring structural pathology [15], and they appear to be largely reversible with weight restoration, although the degree of recovery of cortical thickness is negatively correlated with age [16]. Adolescents with restricting-type anorexia nervosa show especially reduced grey matter volumes in the frontal lobes and insula [56].

The brain’s structural white matter connectivity can be examined with diffusion magnetic resonance imaging techniques, such as diffusion tensor imaging [57,58]. Because structural connectivity underpins functional connectivity, which reflects how different brain regions communicate [59], alterations in white matter networks may contribute to the atypical functional brain patterns observed in AN. Although evidence remains limited, one study found that patients with AN showed increased white matter connectivity in frontal tracts and reduced connectivity in subcortical networks compared with healthy controls [60].

These structural observations are consistent with functional neuroimaging findings in individuals with AN, where studies have demonstrated excessive activation in regions of the frontal cortex, implicated in cognitive control. For example, individuals recovered from AN showed altered frontal activation in response to reward-related stimuli compared to healthy controls [61], suggesting that restrictive behaviour may be driven by pronounced top–down regulation of reward cues. In the mentioned study, reward-related activity was induced using a modified version of the Monetary Incentive Delay Task, a commonly used paradigm for examining reward anticipation processing [62]. Similarly, during tasks requiring behavioural response shifting, AN patients displayed impaired cognitive-behavioural flexibility accompanied by predominant prefrontal activation [63]. Together, these findings support the hypothesis that individuals with AN exhibit excessive recruitment of prefrontal networks, which may contribute to cognitive inflexibility and rigid behaviour.

The imbalance between top–down and bottom–up cortical processes is also reflected in the delay discounting task. Underweight AN patients tend to show greater tolerance for delayed rewards [64]. However, this excessive self-control in the context of delayed rewards appears to be associated with altered activity in cingulostriatal circuitry, particularly in the striatum and dorsal anterior cingulate cortex, rather than increased activity in the prefrontal cortex.

Likewise, fMRI paradigms using food-related visual stimuli often show a pattern of hyperactivation in regions involved in cognitive control and, in some cases, emotional processing [65], together with hypoactivation in regions involved in reward-related processing [66].

Complementing task-based paradigms, resting-state fMRI (rs-fMRI) provides additional support for connectivity alterations in AN. A systematic review of resting-state functional magnetic resonance imaging (rs-fMRI) studies in patients with anorexia nervosa (both current and recovered AN) has provided preliminary evidence of altered functional connectivity in networks involved in cognitive control, flexibility and the processing of body signals, such as visual and somatosensory integration [67]. In particular, regions involved in body imaging processing have been associated with decreased functional connectivity [68]. Furthermore, increased activation within fronto-striatal circuits has been linked to the maintenance of restrictive eating behaviours in AN [69].

### fMRI and psilocybin

The psychedelic experience and state remain somewhat elusive, reflecting the current state of our understanding of consciousness [70]. Nevertheless, findings indicate that psychedelics and their effect on the serotonin signalling system have been associated with change in standard brain network communication, thus change in functional connectivity [71]. Among other things, these changes are thought to influence how the brain interprets the world and facilitate new learning by attributing greater significance to sensory inputs, thereby enhancing the probability of “belief updating” through reduced prediction error [72–74].

Similarly, psilocybin in the acute state has been shown to reduce brain activity in key brain network regions during resting-state, with the extent of this reduction predicting the intensity of subjective psychedelic effects [75]. The reduced brain activity in default network regions is hypothesised to facilitate a state of cognition free from ordinary bias, patterns and rules.

In patients with major depressive disorder, psilocybin has further been associated with a sustained reduction in brain network modularity three weeks post-treatment, and changes in modularity were associated with improvements in depressive symptoms [76]. Moreover, increased dynamic network flexibility was observed in the executive network (EN) and in regions with high in 5-HT2A-receptor density, that are functionally connected to the EN. Notably, psilocybin disrupts connectivity in the default mode network by causing desynchronization across spatial scales [77].

Beyond functional changes, psilocybin may also affect structural connectivity, as suggested by animal studies and emerging human evidence [78,79]. Also, psilocybin has been shown to affect the fronto-striatal-thalamic circuits reducing top-down regulation and enhancing bottom-up information, an effect attributed to decreased influence of structural conditions, hypothesised to be mediated by serotonin and dopamine signalling [80, preprint].

Overall, emerging evidence highlights how psychedelics may benefit AN patients, such as enhanced serotonin signalling and cognitive flexibility [49]. However, further research is needed to elucidate long-term effects, particularly in clinical context. Functional magnetic resonance imaging (fMRI) has demonstrated utility in detecting neuronal abnormalities in AN. This study’s use of fMRI before and after psilocybin treatment may provide insights into the neurobiological impacts of psilocybin on AN.

### Brain-derived neurotrophic factor and AN

Brain-derived neurotrophic factor (BDNF) is a neurotrophin involved in synaptic plasticity, neuronal survival, dendritic growth, and activity-dependent learning processes. It is involved in long-term potentiation and experience-dependent neural remodelling, particularly within cortico-limbic and fronto-striatal circuits that support reward learning, emotional regulation, and cognitive flexibility [81,82].

AN can be conceptualized as a disorder characterized by maladaptive learning and habit formation, in which initially goal-directed weight-control behaviours become rigid, compulsive, and resistant to updating despite negative physiological consequences [83,84]. Altered reward processing, impaired fear extinction, and heightened cognitive control are associated with illness persistence.

Peripheral BDNF concentrations have been reported to be reduced in individuals with acute AN, although findings remain heterogeneous across studies and sampling methods. BDNF levels are also associated with nutritional status and illness severity [85,86]. Longitudinal studies suggest partial normalization of BDNF levels with weight restoration. Although the relationship between peripheral BDNF and central nervous system activity is complex and not fully established, these observations are consistent with the hypothesis that prolonged undernutrition may influence neuroplasticity-related signalling pathways relevant for AN recovery. Given that adolescence represents a period of heightened neurodevelopmental plasticity [14,87], alterations in BDNF signalling during this developmental window may influence illness chronicity and recovery trajectories. In the context of eating behaviour, evidence also indicates an anorexigenic role of BDNF/TrkB signalling in hypothalamic circuits, linking BDNF to body weight regulation [85,86].

### BDNF and psychedelics

Preclinical and translational research suggests that serotonergic psychedelics, including psilocybin, engage neuroplasticity-related pathways [39,88]. Through agonism at the 5-HT2A receptor, psilocybin has been shown to promote dendritic spine growth, synaptogenesis, and increased expression of plasticity-associated genes, including BDNF [50,89,90]. Although the precise relationship between peripheral BDNF changes and clinical outcomes in humans remains under investigation, converging data support the hypothesis that psilocybin may temporarily enhance neural plasticity, potentially facilitating belief updating, emotional learning, and cognitive flexibility during a transient period of increased plasticity [38].

Given the proposed role of maladaptive learning and rigid habit formation in AN, and the emerging evidence that psilocybin may modulate neuroplasticity-related pathways, longitudinal assessment of peripheral BDNF in psiAN provides an exploratory biomarker of treatment-related plasticity. Measuring BDNF alongside clinical outcomes and psychological process measures may help clarify whether changes in neuroplasticity markers are associated with symptom improvement, relapse trajectories, or subjective psychedelic experiences. While peripheral BDNF cannot be interpreted as a direct measure of central synaptic remodelling, its inclusion may contribute to mechanistic insight.

### Adolescents and psychedelic research

AN most commonly emerges during adolescence, with peaks in incidence occurring in mid-to-late teenage years and early adulthood [3]. Early illness trajectories are often marked by rapid consolidation of maladaptive patterns, and longer duration of untreated illness is associated with poorer outcomes [2,7,8]. Despite this, adolescents are frequently excluded from trials of novel psychiatric interventions, including psychedelic research, due to ethical and regulatory concerns. Inclusion of adolescents in clinical research generally requires sufficient evidence of safety and efficacy in the adult population [91–95]. Recent ethical analyses argue that developmental vulnerability alone should not justify categorical exclusion, particularly when existing treatments are insufficient and when safeguards for informed consent, monitoring, and risk minimization are in place. Moreover, exclusion of adolescents from research is not ethically neutral, particularly in disorders such as AN that commonly emerge during adolescence and where delayed access to effective intervention may contribute to chronicity.

While these preclinical findings highlight the importance of developmental caution, observational data in humans indicate that psychedelic use among adolescents and young adults occurs outside clinical settings and when controlling for confounders is not associated with increased rates of psychosis or suicidality at the population level [96,97]. These findings support the importance of generating controlled safety data rather than leaving this population unstudied. Preclinical evidence from a developmental neuroimaging study in mice exposed to psilocybin during adolescence demonstrated persistent changes in brain volume and functional connectivity that extended into adulthood, alongside altered sensory processing, with structural effects more pronounced in males than in females [98]. While cross-species extrapolation requires caution, these findings reinforce the importance of systematic developmental monitoring and support the stepwise safety design adopted in the present protocol.

The present study therefore includes individuals aged 16–35 years with anorexia nervosa who have experienced at least one relapse, a group at high risk for chronic illness with limited treatment options. By adopting a cautious, stepwise design with enhanced monitoring and staged inclusion of minors, this trial aims to address both scientific and ethical imperatives in advancing care for a severely affected population.

## Method

### Study design

The psiAN study is a phase IIa, open-label, randomized controlled clinical trial evaluating the safety, tolerability, and preliminary therapeutic potential of psilocybin administered with structured and manualised psychological support alongside treatment as usual (TAU) in adolescents and young adults with ongoing AN who have previously achieved at least partial weight restoration (BMI ≥ 17) followed by subsequent weight loss, consistent with relapse. Participants are randomized in a 1:1 ratio to either two 25 mg doses of psilocybin treatment plus TAU, or TAU alone. Where discontinuation of interacting medications is required, a supervised washout period is implemented prior to psilocybin administration, based on the pharmacokinetic properties of the medication, clinical assessment, and patient safety considerations.

At 6-month follow-up, the participants of the control arm are offered psilocybin treatment.

The trial includes repeated assessments from baseline through 12 months of follow-up, enabling evaluation of short-term effects and longer-term relapse-related outcomes. Neurobiological measures, including fMRI and BDNF, are integrated to explore potential mechanisms associated with treatment response.

### Study setting

All clinical procedures, including screening, psychological support sessions, and psilocybin administrations, are conducted at the Department of Psychiatry, Lund (Skåne) University Hospital, Lund, Sweden.

Functional magnetic resonance imaging assessments are performed at the National 7T Facility in Lund. The study is conducted in accordance with the Declaration of Helsinki, Good Clinical Practice (GCP), and the EU Clinical Trials Regulation (CTR No. 536/2014), with oversight by relevant national regulatory authorities.

### Participants

Eligible participants are adolescents and young adults aged 16–35 years with a DSM-5 diagnosis of AN who have experienced at least one period of partial weight restoration (BMI ≥ 17) followed by subsequent weight loss consistent with relapse. Participants must have a body mass index (BMI) greater than 16.0 at inclusion and be in stable contact with specialized psychiatric care in Region Skåne, Sweden. Key exclusion criteria include current or lifetime psychotic or bipolar disorder, substance use disorder, clinically significant medical or cardiovascular conditions that contraindicate psilocybin administration or MRI procedures, pregnancy or breastfeeding, and ongoing use of medications with known adverse interactions with serotonergic psychedelics that cannot be safely discontinued.

Concomitant medication management is guided by clinical judgment and current evidence. Ongoing treatment with selective serotonin reuptake inhibitors (SSRIs) and serotonin–noradrenaline reuptake inhibitors (SNRIs) may be continued when clinically appropriate and safe, consistent with emerging evidence suggesting that concomitant SSRI use does not invariably preclude therapeutic response to psilocybin [99,100]. Medications with clinically relevant 5-HT2A receptor antagonistic properties, including antipsychotics, are exclusion criteria unless they can be safely discontinued prior to dosing.

A full list of exclusion criteria can be found in the study protocol.

### Recruitment and informed consent

Participants are recruited through clinicians within specialized eating disorder services in Region Skåne, Sweden. All potential participants receive comprehensive written and verbal information about the study, including potential risks, benefits, and the voluntary nature of participation. Two weeks are provided for consideration from consent to randomization.

For participants aged 18 years and older, written informed consent is obtained directly. For participants aged 16–17 years, written informed consent is obtained from both the participant and both their legal guardians. Consent procedures for minors emphasize developmentally appropriate communication and assessment of understanding.

### Study status

Participant recruitment is ongoing since 2026-03-01. Recruitment is expected to be completed by October 2027. Data collection, including 12-month follow-up assessments for all participants, is anticipated to conclude by October 2028. Results are expected to be available for analysis and dissemination from 2029 onwards.

### Patient and public involvement

Prior to protocol finalisation, focus groups were conducted with individuals with lived experience of eating disorders to inform the trial design. Participants highlighted the need for enhanced clarity and adequate advance notice in study procedures, which led to revision of participant and next-of-kin information materials to improve comprehensibility and support informed decision-making. Focus group data will not be published.

### Randomization and allocation

Participants are randomized using a computer-generated block randomization sequence in a 1:1 ratio to either psilocybin treatment plus TAU or TAU alone. Randomization is stratified by age group (16–17 years and 18–35 years) to ensure balanced representation. Allocation is conducted by personnel independent of the research team. Due to the nature of the intervention and the pronounced subjective effects of psilocybin, blinding is not employed in this study.

### Interventions

#### Psilocybin therapy.

Participants allocated to the treatment arm receive two oral administrations of psilocybin at a fixed dose of 25 mg, administered four weeks apart, in addition to TAU. Psilocybin is administered in a controlled clinical environment within a manualized structure of psychological support delivered by two trained therapists, including preparatory sessions prior to dosing, non-directive support during dosing, and integration sessions following each administration. Psychological support follows established safety guidelines for psychedelic research and is tailored to the specific vulnerabilities associated with anorexia nervosa.

#### Psychological support.

Psychological support is an integral component of the active treatment and is provided to all participants receiving psilocybin in addition to treatment as usual. The psychological support is delivered by two therapists with experience in the treatment of eating disorders and who have completed five days of training in the method. The approach is non-directive and supportive rather than interpretative, with the primary aim of fostering psychological safety, openness to experience, and integration of experiences within the participant’s ongoing clinical context.

Psychological support is manualized and organized into three phases. It includes two 1-hour preparatory sessions before the first psilocybin administration, with an additional preparatory component incorporated into the third integration session before the second dosing session; supportive therapist presence throughout both full-day dosing sessions; and post-session integration consisting of three 1-hour sessions after the first dosing session and two 1-hour sessions after the second. Preparatory sessions focus on building therapeutic alliance, clarifying intentions, discussing expectations, and preparing participants to navigate challenging experiences. During dosing sessions, therapists provide continuous support, emphasizing reassurance, grounding, and acceptance of emerging experiences without attempting to guide content or outcomes. Integration sessions following each dosing focus on helping participants reflect on and contextualize their experiences, relate insights to everyday life and recovery goals, and support adaptive behavioural and emotional changes. If additional integration support is deemed clinically necessary by the PI, protocol-defined booster sessions may be offered and documented for transparency.

The psiAN psychological support model is tailored to the vulnerabilities associated with AN, including fear of loss of control, and sensitivity to bodily and emotional experiences as well as considerations related to food intake on dosing days. Therapists maintain close coordination with participants’ regular treatment providers to ensure continuity of care and appropriate clinical follow-up. Psychological support is manualized to ensure consistency across participants while allowing flexibility to meet individual needs and uphold safety throughout the study. The research team actively visits referring units to inform and educate the regular care providers, including organizing onsite in Lund and online psilocybin therapy education days twice a year during the duration of the study.

A limitation of the design is that participants in the TAU arm do not receive matched therapist contact, which may contribute to nonspecific between-group differences.

#### Support person.

Participants in the psiAN study are required to identify a support person who is available at all sessions for support. This person may be a parent, guardian, family member, partner, or close friend and is informed about the study procedures, expected variations in effects, and post-session recommendations. While not present during dosing, the support person is asked to provide practical and general emotional support in the immediate post-session period. In the first preparation visit the therapists give clear instructions about which sessions the support person is included actively, and which sessions are only for the participant, and encourage sharing of own questions and experiences in relation to the study.

The inclusion of a support person is grounded in established evidence from anorexia nervosa (AN) treatment research. Family-based therapy (FBT), the first-line treatment for adolescents with AN, emphasizes caregiver involvement as central to recovery and relapse prevention [2,101]. Even in adult populations, social support and close relational involvement are associated with improved treatment adherence and outcomes [10]. Given the high relapse rates and medical vulnerability associated with AN, structured involvement of a trusted support figure may enhance safety, reinforce behavioural change, and buffer against deterioration during transitional phases of care.

In psychedelic-assisted therapy, the importance of interpersonal context, commonly conceptualized as “set and setting,” is well established. Supportive interpersonal environments are associated with reduced risk of adverse psychological reactions and improved integration of acute psychedelic experiences [72,102]. Contemporary clinical trials have emphasized the role of structured psychological support and stable post-session environments in optimizing safety and therapeutic benefit [103]. The presence of a designated support person following dosing aligns with this framework by extending containment and emotional scaffolding beyond the clinical setting.

The inclusion of a support person is particularly important for adolescents. Adolescence is characterized by ongoing neurodevelopment, heightened emotional sensitivity, and increased reliance on caregiver support for regulation and decision-making [104]. Ethical frameworks for research involving minors therefore emphasize enhanced protective measures such as guardian engagement and environmental stability. In eating disorders, early caregiver involvement is strongly associated with better long-term outcomes and reduced chronicity [2]. Within the context of psychedelic-assisted therapy—where experiences may be emotionally intense or personally meaningful—structured support from a trusted adult may mitigate risk, facilitate integration, and support adherence to recovery-oriented behaviours.

The requirement of a support person in psiAN reflects evidence-based principles from eating disorder treatment, psychedelic clinical research, and adolescent developmental psychiatry. It represents a protective and ethically responsive design feature intended to enhance safety, promote integration, and support sustained recovery.

#### Treatment as usual.

Participants in both study arms continue to receive treatment as usual (TAU), consisting of evidence-based, multidisciplinary psychiatric care for eating disorders delivered at psychiatric clinics in Region Skåne, Sweden. TAU may include psychotherapy, physiotherapy, nutritional rehabilitation, medical monitoring, and psychosocial support. The TAU teams are actively involved in the study and constitute the primary recruitment pathway. Twice a year, all TAU teams in Region Skåne are invited to a full-day briefing hosted by the research team, providing study updates and relevant scientific information. Between these meetings, the research team maintains ongoing communication with TAU units to support recruitment and to promote consistent, best-practice clinical management of participants receiving psilocybin therapy alongside TAU. This collaboration is intended to facilitate continuity of care, integration of treatment-related experiences, and being aware of possible adverse events related to psilocybin and knowing how to report to the research team.

### General analysis strategy

#### Primary outcome.

The primary outcome of the psiAN trial is safety and tolerability of psilocybin therapy in adolescents and young adults with relapsing AN measured at primary endpoint compared with TAU. Safety is evaluated through systematic monitoring of adverse events (AEs) and serious adverse events (SAEs) from the time of intervention initiation through 12-month follow-up. Cardiovascular safety is assessed using electrocardiography and repeated measurements of blood pressure and pulse. Laboratory monitoring includes glucose, liver and kidney function tests, and urine toxicology. Physical stability is evaluated through longitudinal measurement of body mass index (BMI). Psychiatric safety is assessed using structured suicidality assessment and general psychiatric symptom ratings at predefined time points. The primary safety evaluation occurs at primary endpoint at week 9, with continued surveillance at month 3,4,5,6 9 and 12 months.

#### Secondary outcomes.

Eating disorder symptom severity is assessed longitudinally, together with BMI, to characterize changes in core psychopathology and weight stability. Time to relapse is evaluated across the 12-month follow-up period. Relapse is operationalized as a composite endpoint defined by clinically meaningful weight loss, rehospitalization for eating disorder–related complications, initiation of intensified treatment or higher level of care, or clinically significant self-reported symptom deterioration confirmed by clinical assessment.

Mood and anxiety symptoms are assessed repeatedly to examine broader psychiatric change. Psychological well-being and affective functioning are evaluated to capture potential improvements beyond symptom reduction. Personality traits are measured to explore baseline moderators and potential trait-level changes associated with treatment. Acute subjective psychedelic experiences are assessed following dosing sessions and examined in relation to subsequent clinical outcomes.

fMRI is conducted longitudinally as an exploratory outcome to characterize potential treatment-associated changes in brain network function and to examine their relationships with clinical outcomes over time. In parallel, peripheral blood samples are collected repeatedly to quantify BDNF as an exploratory marker of neuroplasticity and to explore associations with symptom change, relapse trajectories, and subjective treatment response.

### Study measures

**Fig 1:**
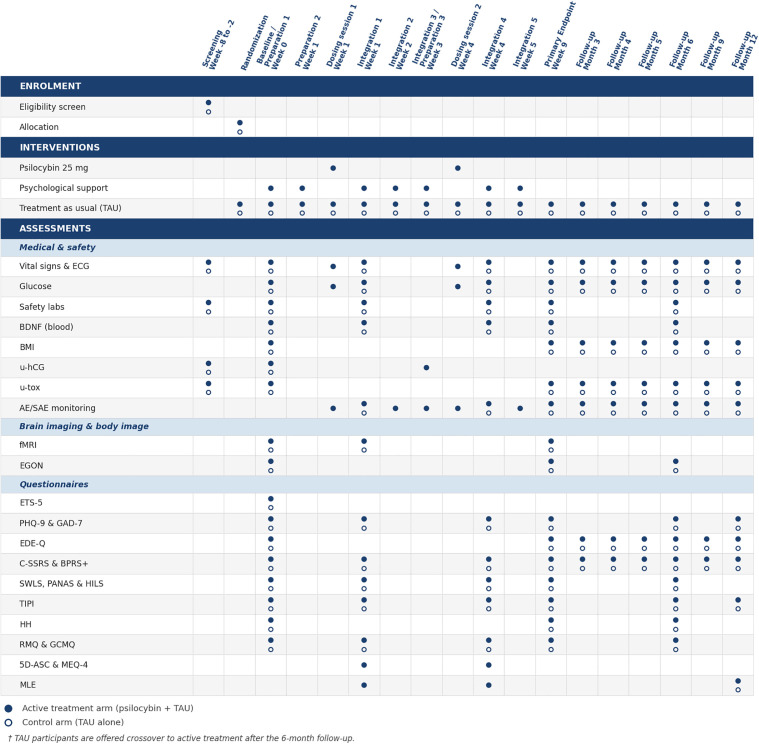
Schedule of assessments. There is a minimum of 2 weeks from consent to randomization, time from screening to the first psilocybin dose must not exceed 8 weeks, regardless of washout status. **Abbreviations:** ECG, electrocardiogram; u-hCG, urine human chorionic gonadotropin; u-tox, urine toxicology screening; fMRI, functional magnetic resonance imaging; BDNF, brain-derived neurotrophic factor; BMI, body mass index; AE/SAE, adverse event/serious adverse event; ETS-5, Expectation of Treatment Scale [105]; RMQ, Readiness and Motivation Questionnaire [106]; GCMQ, General Change Mechanisms Questionnaire [107]; PHQ-9, Patient Health Questionnaire-9 [108]; GAD-7, Generalized Anxiety Disorder-7 [109]; EDE-Q, Eating Disorder Examination Questionnaire [110]; C-SSRS, Columbia-Suicide Severity Rating Scale [111]; BPRS + , Brief Psychiatric Rating Scale–Extended [112]; SWLS, Satisfaction with Life Scale [113]; PANAS, Positive and Negative Affect Schedule [114]; HILS, Harmony in Life Scale [115]; TIPI, Ten Item Personality Inventory [116]; HH, Honesty-Humility Scale [117]; 5D-ASC, Altered States of Consciousness Rating Scale [118]; MEQ-4, Mystical Experience Questionnaire [119]; MLE, Meaningful Life Experience Rating [120]; EGON-instrument, clinical assessment tool for evaluating body perception [121].

Study measures are selected to comprehensively assess safety, clinical outcomes, psychological processes, subjective experience, and neurobiological correlates of change from baseline, across the acute, post-acute, and long-term follow-up phases of the trial. The timing of all assessments is summarized in the Schedule of Assessments (Fig 1).

#### Safety and tolerability measures.

Safety and tolerability constitute the primary outcome domain and are monitored throughout the study from first dosing to 12-month follow-up. Medical safety assessments include electrocardiography (ECG), blood pressure (BP), pulse, blood tests including glucose, liver and kidney function and urine toxicology (U-tox). Pregnancy testing is conducted at screening and prior to each dosing session.

Adverse events (AEs) and serious adverse events (SAEs) are recorded continuously from first dosing through the 12-month follow-up. Psychiatric safety is assessed using the Columbia–Suicide Severity Rating Scale (C-SSRS) [111] and the Brief Psychiatric Rating Scale–Expanded (BPRS+) [112], enabling systematic monitoring of suicidality, psychotic symptoms, affective instability, and general psychiatric deterioration across study phases.

#### Eating disorder symptomatology.

Eating disorder symptom severity is assessed using the Eating Disorder Examination Questionnaire (EDE-Q) [110]. The EDE-Q evaluates restraint, eating concern, shape concern, and weight concern, providing both global and domain-specific indices of psychopathology. Body mass index (BMI) is measured longitudinally to monitor weight stability and detect clinically meaningful weight change.

Relapse is operationalized as a composite outcome defined by clinically significant BMI decline, rehospitalization for eating disorder–related complications, initiation of intensified treatment or higher level of care, or clinically significant self-reported symptom deterioration confirmed by clinical assessment.

The EGON instrument is administered at baseline, primary endpoint, and six-month follow-up. EGON is a multidimensional assessment tool designed to evaluate body perception in clinical settings. One of its primary components involves participants estimating nine body dimensions, which are compared with objective body measurements. This comparison is expressed as the Body Perception Index (BPI), reflecting the degree to which individuals overestimate or underestimates their body size. This clinical developed tool builds on principles from the method described by Slade and Russell [121].

#### General psychopathology and well-being.

Depressive symptoms are assessed using the Patient Health Questionnaire-9 (PHQ-9; [108]), and anxiety symptoms are assessed using the Generalized Anxiety Disorder-7 (GAD-7; [109]). These measures capture common comorbid symptom domains in anorexia nervosa and allow assessment of broader psychiatric changes associated with the treatment. Psychological well-being and affective functioning are assessed using the Positive and Negative Affect Schedule (PANAS; [114]), the Satisfaction with Life Scale (SWLS; [113]), and the Harmony in Life Scale (HILS; [115]). Together, these instruments assess emotional valence, life satisfaction, and existential coherence.

#### Psychological process measures.

Treatment expectancy is measured at baseline using the 5-item version of Expectation of Treatment Scale (ETS-5; [105]). Motivation and readiness for change are assessed using the Readiness and Motivation Questionnaire (RMQ; [106]) at baseline and repeated post-dosing time points. The General Change Mechanisms Questionnaire (GCMQ; [107]) is administered during integration phases to assess perceived cognitive, emotional, and behavioural change processes associated with therapeutic progress.

Personality traits are assessed using the Ten Item Personality Inventory (TIPI; [116]) and the Honesty–Humility scale (HH; [117]), allowing exploration of baseline moderators and potential trait-level changes over time.

#### Subjective psychedelic experience.

Acute subjective effects of psilocybin are assessed using the Altered States of Consciousness Rating Scale (5D-ASC; [118]), the Mystical Experience Questionnaire (MEQ-4; [119]), and the Meaningful Life Experience rating with a single item (MLE; [120]). These instruments quantify experiential intensity, mystical-type experiences, and perceived personal significance, enabling examination of associations between acute subjective phenomena and subsequent clinical or biological outcomes.

#### Neuroimaging.

MRI will be performed at three time points: at baseline, the day after the first dosing session and at primary endpoint (week 9). The scans will be acquired on a 7 Tesla Philips Achieva MRI system (Philips Medical Systems, Best, The Netherlands) with a 32-channel head coil. The MRI examination will include an anatomical image using magnetization-prepared rapid acquisition gradient-echo (MP-RAGE) for T1-weighted imaging. The examination will thereafter include diffusion tensor imaging (DTI) to estimate structural connectivity, followed by resting-state fMRI (rs-fMRI) measurement in line with previous psilocybin research to assess global connectivity measures. Two task-based fMRI paradigms will then be conducted: a simplified version of the Monetary Incentive Delay task (MIDT) [62,122] and a Calorie-Cue Task (CCT) similar to previous food-picture paradigms used in research on AN [65,123]. MIDT will evaluate striatal activity during reward anticipation and CCT will assess differentiation of limbic and prefrontal activation during high-, low and no calorie cues.

The simplified version of the MIDT implemented will focus on reward anticipation only (not anticipation of loss). Participants will view cues indicating either a high (5 SEK) or low reward (0.1 SEK). A subsequent target will require a rapid response, with the response window adjusted individually to achieve a reward rate of approximately 75–80%. fMRI blood-oxygen-level-dependent (BOLD) signals will be acquired during the anticipation phase. Analyses focus on striatal activity, with the low-reward condition serving as a baseline.

CCT is an in-house developed food-picture paradigm alternating pictures presenting images of female individuals consuming high-calorie food, low-calorie food and holding a neutral item (no calorie). Each condition is presented in 20-second blocks, defined as one sequence. After each sequence the participants rate the unpleasantness of the image on a scale from 0 to 10 followed by a 15-second rest period. A total of six sequences is presented, with two sequences per condition. This design will enable assessment of differences in BOLD responses between calorie-dependent conditions, adjusted for subjective unpleasantness ratings.

#### BDNF.

Peripheral BDNF will be measured longitudinally within psiAN, with a particular focus on detecting post-dosing changes relative to baseline. Blood sampling will be scheduled to 3:00 pm the day after 25 mg psilocybin administrations.

BDNF is stored in human platelets and can be released upon platelet activation, meaning that serum BDNF largely reflects platelet-derived BDNF released during coagulation, resulting in high concentrations and substantial method-dependent variability [124]. Serum concentrations are strongly influenced by clotting conditions, including clotting time, whereas plasma measurements are influenced by pre-analytical handling and centrifugation strategy. For detecting time-sensitive effects, EDTA plasma is preferred because it more closely reflects the circulating, non–platelet-mediated pool and is therefore more suitable for tracking acute changes in BDNF following pharmacological interventions. Consistent with best-practice recommendations for improving reproducibility, platelet-poor plasma (PPP) will be prepared using a double-centrifugation approach.

Limited evidence from psychedelic biomarker studies shows that low doses of LSD acutely increase BDNF in plasma within 4–6 hours in a placebo-controlled within-subject design, indicating that plasma can detect rapid post-dose changes in circulating BDNF [125]. Although psiAN sampling occurs within a broader window (~24 h), use of plasma and strict pre-analytical standardization is intended to maximize sensitivity for acute effects. In eating disorder populations, peripheral BDNF is commonly reported to be lower than in controls, and a systematic review/meta-analysis found no strong basis to prefer serum over plasma solely because of group differences.

Blood will be processed to platelet-poor plasma using double centrifugation within 60 minutes of sampling. Processing time, centrifugation parameters, and any deviations will be documented. Plasma aliquots will be frozen as soon as possible after processing and stored at −80°C until batch analysis. This standardized workflow is intended to minimize pre-analytical variance and improve comparability across time points and participants.

BDNF will be quantified using the MSD U-PLEX Human BDNF assay, which is validated for both EDTA plasma and serum and supports standardized dilution procedures. The assay will be performed in accordance with manufacturer recommendations and internal laboratory quality procedures, including appropriate calibration curves, controls, and documentation of sample handling.

To explore whether genetic variation in BDNF signalling moderates biomarker trajectories or clinical outcomes, participants will be genotyped for the BDNF Val66Met polymorphism (rs6265) [85]. This variant has been associated with altered activity-dependent BDNF secretion and has been discussed as a potential moderator of neuroplasticity-related phenotypes. Genotyping will be performed from whole blood collected at baseline using standard genotyping methods for analysis. Val66Met status will be used in exploratory analyses as a potential moderator of baseline BDNF levels, acute post-dosing BDNF change, and associations with clinical outcomes.

#### Long-term follow-up assessments.

Participants are followed for up to 12 months after baseline to assess durability of effects and relapse-related outcomes. Follow-up assessments include repeated evaluation of eating disorder symptoms, psychiatric safety, adverse events, medical parameters, and selected psychological measures. This extended follow-up period allows examination of both sustained benefits and delayed adverse effects, which is particularly important in early-phase trials involving adolescents and young adults.

### Data management

All data are collected by GCP-trained study personnel and entered a secure electronic case report form system (REDCap) compliant with data protection regulations. Data are pseudonymized by a code list with access restricted only to authorized personnel delegated by the principal investigator. All data are stored and processed using secure institutional infrastructure at Lund university, Sweden. Anonymized participant data will be made available upon reasonable written request to the principal investigator, contingent on adherence to a conventional data-sharing agreement and compliance with all applicable ethical and regulatory requirements.

### Statistical analysis

Analyses are primarily descriptive and hypothesis-generating, reflecting the pilot nature of the study. Safety outcomes are summarized using frequencies and proportions, with between-group comparisons performed using chi-square or Fisher’s exact tests as appropriate. Longitudinal clinical outcomes are analysed using mixed-effects models to account for repeated measures and individual variability. Time-to-event outcomes, including relapse, are analysed using survival analysis methods.

Neuroimaging analyses focus on within-subject changes from baseline and between-group contrasts at predefined time points. Associations between neuroimaging measures, clinical outcomes, and subjective experience variables are explored using correlational and regression-based approaches. Appropriate correction for multiple comparisons is applied, particularly for neuroimaging analyses. Missing data are handled using maximum likelihood estimation or multiple imputation, depending on the pattern and extent of missingness.

### Sample size

No formal power calculation has been performed, as the study is designed as an early-phase trial focused on safety, feasibility, and estimation of effect sizes. The planned sample size of 40 participants is based on feasibility considerations and consistency with prior early-phase psychedelic studies in clinical populations to be able to detect adverse events. The six-month follow-up invites TAU participants for a switch to active treatment protocol, therefore leaving the active treatment arm without control until the 12-month follow-up and end of study.

### Participant safety and monitoring

Participant safety is the primary focus of the psiAN trial and is ensured through structured medical and psychiatric screening, continuous monitoring during dosing, repeated short- and long-term follow-up assessments, and independent safety oversight. Attention is given to metabolic stability given the medical vulnerability associated with anorexia nervosa. Nutritional status is assessed at screening and baseline, and participants must meet predefined BMI of 16 and medical stability criteria prior to dosing. Preparation sessions include structured discussion of food intake, hydration, and individualized eating plans for the dosing day to minimize risk of hypoglycaemia. Blood glucose is measured at screening and monitored in accordance with protocol-defined procedures.

This approach is informed by prior psilocybin research in anorexia nervosa, in which an isolated case of transient hypoglycaemia was reported during dosing in a medically stable participant and resolved with oral intake and monitoring [53]. To mitigate similar risks, psiAN incorporates proactive nutritional planning, pre-dose fasting guidance tailored to medical status, real-time clinical monitoring, and availability of oral glucose and medical support during dosing sessions. Adverse events and serious adverse events are recorded continuously from first dosing through the 12-month follow-up and reported in accordance with regulatory requirements. Suicidality is systematically assessed at screening, prior to dosing, during integration, at the primary endpoint, and at all follow-ups. Participants demonstrating medical instability or acute psychiatric risk are withdrawn from dosing and referred for appropriate clinical care.

An independent Safety Review Committee evaluates accumulating safety data at predefined intervals. Recruitment begins with adults (≥18 years), and inclusion of adolescents aged 16–17 years proceeds only after formal safety review and approval, with enhanced monitoring and guardian involvement.

Participants may withdraw at any time, and investigators may discontinue participation if safety concerns arise.

### Ethical considerations

This study has received approval from the Swedish Ethical Review Authority (Dnr 2025-05495-01) and the Swedish Medical Products Agency and is conducted in accordance with the Declaration of Helsinki, Good Clinical Practice (GCP), and the EU Clinical Trials Regulation (EU No. 536/2014). Given the vulnerability of the study population and the inclusion of a psychedelic intervention, extensive safeguards are implemented to minimize risk and ensure participant well-being.

In addition to the monitor, a Safety Review Committee is recruited to oversee trial conduct and review all potential serious adverse events, protocol adherence, and emerging safety signals. Interim safety reviews are conducted after predefined numbers of psilocybin administrations. Recruitment initially includes only participants aged 18 years and older. Inclusion of adolescents aged 16–17 years is initiated only after the Safety Review Committee has completed a formal evaluation of safety and tolerability data from the initial adult cohort and has approved progression to the adolescent phase.

Protocol amendments are managed in accordance with EU Clinical Trials Regulation 536/2014. Substantial amendments are submitted to and require approval by the competent authority via CTIS (Clinical Trials Information System) prior to implementation. Non-substantial amendments are documented in a note-to-file and submitted alongside the next substantial amendment or as part of annual reporting, as applicable. All approved amendments are communicated to the trial monitor and the full research team without delay.

The stepwise inclusion of minors reflects a balance between ethical caution and the need to generate clinically relevant evidence. The staged design ensures that exposure of adolescents to psilocybin occurs only after safety has been demonstrated in adults within the same protocol framework. Additional safeguards for minors include guardian involvement in the consent and treatment process and age- appropriate information.

Participants may withdraw from the study at any time without consequences for their standard treatment. Procedures are in place for rapid clinical intervention in the event of medical or sustained psychological deterioration. The ethical design of the psiAN study aims to balance the obligation to protect vulnerable individuals with the responsibility to generate evidence for populations with substantial unmet clinical need.

### IMP management

The investigational medicinal product (IMP) is GMP-manufactured psilocybin produced by Filament Health (Vancouver, Canada) and supplied as encapsulated 25 mg oral doses with accompanying batch certification.

EU distribution and release are coordinated by CEFEA in Poland, and importation into Sweden is managed by Oriola in accordance with applicable regulatory requirements. Upon arrival, the IMP is received, stored, and dispensed by the Region Skåne Hospital Pharmacy.

Full traceability is maintained through documented receipt, storage, dispensing, reconciliation, and destruction procedures in accordance with the EU Clinical Trials Regulation and Swedish national regulations.

### Dissemination

The findings of the psiAN study will be disseminated through publication in peer-reviewed scientific journals and presentation at national and international conferences in psychiatry, eating disorder research, and psychedelic science. Results will be reported regardless of outcome, in accordance with principles of transparency and responsible research conduct.

In addition to academic dissemination, study findings will be communicated to broader audiences through public lectures, open presentations, and engagement with professional and community organizations. Where appropriate, results may be shared through media channels to promote accurate and responsible public understanding of psychedelic research in eating disorders. Communication will be conducted in a balanced and evidence-based manner, emphasizing both potential benefits and limitations.

Study outcomes will also be shared with participating clinical services and summarized in accessible formats for participants and individuals with lived experience. Trial registration details and key study information will remain publicly available through relevant clinical trial registries in compliance with regulatory requirements (ClinicalTrials.gov Identifier: NCT07169747).

Data reporting will follow established guidelines for clinical trials to promote methodological transparency and facilitate replication. Any secondary analyses will be clearly identified as exploratory.

## Conclusion

The psiAN study addresses a gap in current treatment options for AN by investigating psilocybin-assisted therapy in a controlled clinical setting with integrated neurobiological assessment. Several features distinguish this protocol: the stepwise inclusion of adolescents aged 16–17 years after a formal adult safety review, the use of 7T fMRI and peripheral BDNF as exploratory mechanistic measures, a manualized psychological support model tailored to AN-specific vulnerabilities, and the structured involvement of existing treatment teams and the family system.

The study has limitations inherent to its early-phase design. The open-label format precludes blinding, and the sample size of 40 participants limits statistical power for secondary outcomes. The absence of an active placebo means that expectancy effects cannot be fully controlled. These constraints are consistent with the primary focus on safety and tolerability, which, given the vulnerability of the population and the novelty of the intervention, supports an open-label design as an appropriate first step.

By publishing this protocol, we aim to enhance transparency and methodological rigour so that the design can be critically appraised and adapted in future trials. The results are expected to provide the first controlled safety data on psilocybin in adolescents and young adults with AN and may clarify how psilocybin-assisted therapy interacts with the clinical and neurodevelopmental features of early-onset eating disorders.

## Supporting information

S1 File psiAN study protocol. (DOCX)
