# Supplementary material for: Study Protocol for “Exploring the safety and therapeutic potential of psilocybin in the treatment of anorexia nervosa in adolescents and young adults”
Source: PLoS One. 2026 Jun 30;21(6):e0352246. doi: 10.1371/journal.pone.0352246 (PMC13318048; doi:10.1371/journal.pone.0352246)
Supplement: S1 File — (DOCX) [file pone.0352246.s001.docx]

**The safety, tolerance and therapeutic potential of psilocybin in anorexia nervosa adolescents and young adults.**

**Sponsor**

**Pouya Movahed Rad**

Lunds University and Region Skåne

**Contact information**

Pouya Movahed Rad (Principal investigator), pouya.movahed_rad@med.lu.se, +46705729613

David Sjöström (Project Coordinator), [david.sjostrom@med.lu.se](mailto:david.sjostrom@med.lu.se), +739075390

EU Trial Number: [2024-515163-63-00](https://euclinicaltrials.eu/ct-sponsor-services/index.html#!/trials/2024-515163-63-00/summary)

Protocol code: psiAN24

Table of Contents

1. Synopsis

2. Background and Rationale

3. Benefit-Risk Evaluation

4. Trial Objectives

- 4.1 Primary Objective

- 4.2 Secondary Objectives

- 4.3 Primary Endpoint

- 4.4 Secondary Endpoint

5. Trial Design and Procedures

- 5.1 Overall Trial Design

- 5.2 Procedures and Flow Chart

- 5.3 Biological Sampling Procedures

- 5.4 Start, End, Temporary Halt, and Early Termination

6. Subject Selection

- 6.1 Inclusion Criteria

- 6.2 Exclusion Criteria

- 6.3 Screening and Inclusion

- 6.4 Withdrawal Criteria

7. Trial Treatments

- 7.1 Description of Investigational Medicinal Products

- 7.2 Auxiliary Medicinal Products

- 7.3 Concomitant Use of Other Medicinal Products and Treatments

- 7.4 Treatment After Trial End

8. Methods for Measurement of Endpoints for Clinical Efficacy and Safety

- 8.1 Methods for Measurement of Endpoints for Clinical Efficacy

- 8.2 Methods for Measurement of Endpoints for Clinical Safety

9. Handling of Adverse Events

- 9.1 Definitions

- 9.2 Assessment of Adverse Events (AE)

- 9.3 Reporting and Registration of Adverse Events

- 9.4 Follow-up of Adverse Events

- 9.5 Independent Data Monitoring Committee

- 9.6 Annual Safety Report (ASR)

- 9.7 Procedures in Case of Emergencies, Overdose, or Pregnancy

10. Statistics

- 10.1 Analysis Population

- 10.2 Statistical Analyses

- 10.3 Adjustment of Significance and Confidence Interval

- 10.4 Sample Size Calculations

- 10.5 Interim Analysis

11. Quality Control and Quality Assurance

- 11.1 Quality Assurance and Sponsor Oversight

- 11.2 Monitoring

- 11.3 Source Data

- 11.4 Deviations, Serious Breaches, and Other Reporting Obligations

- 11.5 Audits and Inspections

12. Ethics

- 12.1 Compliance to the Protocol, ICH-GCP, and Regulations

- 12.2 Ethical Review of the Trial

- 12.3 Procedure for Obtaining Informed Consent

- 12.4 Data Protection

- 12.5 Insurances

13. Substantial Changes to the Trial

14. Collection, Handling, and Archiving of Data

- 14.1 Case Report Form (CRF)

15. Notification of Trial Completion, Reporting, and Publication

16. References

17. Attachments

1. Synopsis

Aim: To explore the safety, tolerability, and initial efficacy of psilocybin administered with psychological support compared to Treatment as Usual (TAU) in adolescents and young adults aged 16-35 with anorexia nervosa (AN).

Primary Objective: To assess the safety and tolerability of psilocybin 25 mg in this population.

Secondary Objectives: To evaluate the efficacy of psilocybin with psychological support in reducing AN symptom compared to TAU, investigate potential mechanisms of action through self-report questionnaires, neuroimaging and BDNF analysis, and conduct qualitative analysis of subjective experiences.

Trial Design: Open, randomized controlled trial with two arms: (1) two doses of psilocybin (25 mg) with psychological support with one month apart and alongside treatment as usual, and (2) Treatment as Usual (TAU).

Trial Population: Adolescents and young adults aged 16-35 diagnosed with anorexia nervosa.

Number of Subjects: 40 participants (20 per group).

Inclusion Criteria: Diagnosed with AN per DSM-5, aged 16-35, BMI >16, who have experienced at least one period of weight gain followed by weight loss.

Exclusion Criteria: Psychosis, bipolar disorder, substance use disorder, contraindicated medications, severe medical conditions, or metal contraindications for MRI.

Intervention: Two doses of 25 mg psilocybin with psychological support, or TAU. Psilocybin sessions will be accompanied by preparatory and integration sessions.

Investigational Medicinal Product Dosage Administration: Psilocybin 25 mg administered orally during supervised sessions.

Ethical Considerations/Benefit-Risk: Comprehensive safety monitoring, support during sessions, and procedures in place for managing any adverse events. First 25 participants included above 18 years of age, followed by 15 patients between 16-17 years of age. Safety review committee after first 10 psilocybin dosing sessions to review adverse events and protocol adherence. Literature on psilocybin clinical trials report low risk for adverse events and large gap in treatment options for patients with relapse in anorexia nervosa.

Planned Duration of the Trial: Q3 2025 – Q2 2027.

2. Background and Rationale

This pilot study will assess the safety, tolerability, and preliminary efficacy of administering 25 mg of psilocybin, spaced one month apart, in combination with treatment as usual (TAU) for adolescents and young adults aged 16-35 with AN. The study will compare this combined approach to the effects of TAU alone. Additionally, it will explore potential mechanisms of action through self-reports, neurobiological assessments, and neuroimaging techniques.

**Anorexia Nervosa**

AN impact approximately 1.0% of older female adolescents and young women. This heterogeneous psychiatric disorder is characterized by significantly low body weight, distorted body image, and an intense fear of weight gain (Hoek, 2006). More than half of adult patients with AN develop a chronic and severe form of the condition (Fichter et al., 2017; Steinhausen, 2002).

AN is known for having the highest mortality rate among psychiatric disorders, being approximately 5 times higher than that of the general population (Arcelus et al., 2011). It also leads to a substantial decline in health-related quality of life (Dejong et al., 2016). Anorexia nervosa (AN) affects multiple organ systems due to severe weight loss and malnutrition, impacting the cardiovascular, gastrointestinal, hematological, and endocrine systems (Westmoreland et al. 2016). Amenorrhea, resulting from disruptions in the hypothalamic-pituitary-gonadal axis, is common, leading to infertility (Puckett et. al. 2021). Low BMI and decreased gonadal function are suggested as key predictors of osteoporosis and fractures in patients with AN (Steinman & Shibli-Rahhal, 2019). In one study, 54% of patients had osteopenia and 21% had osteoporosis in the lumbar spine within 3-4 years of diagnosis ([Zipfel](https://www.researchgate.net/profile/Stephan-Zipfel?_tp=eyJjb250ZXh0Ijp7ImZpcnN0UGFnZSI6InB1YmxpY2F0aW9uIiwicGFnZSI6InB1YmxpY2F0aW9uIn19) et al., 2002).

The age of onset for AN typically shows a bimodal distribution, peaking at 14 and 18 years of age (Halmi et al., 1979). Research indicates various psychological factors, including difficulties with affect regulation, dichotomous thinking, mental rigidity, and a limited capacity for mentalization, might contribute to the persistence of severe chronic AN (Halmi, 2013).

Different psychotherapies have shown to be somewhat effective in the treatment of AN, one review including 5590 patients showed 46% recovered AN patient within 4 years, however 20% remained chronically ill (Steinhausen, 2002). Earlier treatment was associated with better chances of recovery. However, in those who recover, the relapse rate can be more than 50% (Khalsa, 2017).

**Psilocybin and psychedelic therapy**

Psilocybin is part of a group of hallucinogenic compounds, primarily acting as agonists of the 5-hydroxytryptamine (5-HT) 2A receptor, induce effects in domains of cognition, emotion, perception and self-awareness (Swanson, 2018). Although limited in size, clinical trials including various psychiatric disorders indicate the potential beneficial effects of psychedelic based interventions, especially with psilocybin (Carhart-Harris and Goodwin, 2017).

Personality changes have been found to persist up to at least one year after a single high dose (30 mg/70kg) of psilocybin (MacLean et al., 2013). Similarly, change in the personality trait after intake of another psychedelic substance, Lysergic-acid-diethylamide (LSD), intake has been linked to increased variability and complexity in brain activity (Lebedev et al., 2016). Although, the therapeutic mechanisms are still mostly unknown (Mertens et al., 2021).

While research on psychedelics is growing, studies focusing on the use, experiences, and effects of these substances among adolescents and young adults remain limited (Bates and Trujillo, 2021). There's an acknowledged need for novel psychiatric treatments for adolescents, like adults, but with additional considerations for developmental stages and informed consent processes (Rajwani, 2022).

**Psychedelics and eating disorders**

Ayahuasca is a psychoactive brew typically consisting of plants containing dimethyltryptamine (DMT), a classic psychedelic substance like psilocybin, inducing altered states of consciousness during ceremonial use. Two qualitative studies involving a total of 29 participants who underwent ayahuasca ceremonies for eating disorders (ED) have been published (Lafrance et al., 2017; Renelli et al., 2020). The studies identified five key themes from the participants' experiences: (1) effectiveness of ayahuasca in reducing AN symptom and aiding overall recovery, (2) deeper awareness of the root causes of ED, (3) processing of intense emotions and memories, (4) insights into love, self-love, and self-care, and (5) a spiritual aspect to their healing and recovery.

Recent reviews suggest mechanisms of change for psychedelics in AN patients, such as increased serotonin system signaling and enhanced cognitive flexibility (Calder et al., 2023; Foldi et al., 2020). The ability of psychedelics to foster cognitive flexibility, a well-documented phenomenon, is considered a key factor in therapeutic processes (Davis et al., 2020; Lebedev et al., 2016; Carhart-Harris et al., 2016b). This is particularly relevant for AN patients, who often exhibit rigid thinking and behavior, a factor contributing to treatment resistance and the development of severe, persistent symptoms (Halmi, 2013). In addition, psilocybin and other classical psychedelics can induce temporary ego-dissolution, potentially offering new perspectives on self-image (Nour et al., 2016; Mason et al., 2020).

Currently, there are no approved pharmacological treatments for anorexia nervosa (Rahkonen et al., 2022). However, three pilot clinical trials have been approved and activated. NCT04052568 from John Hopkins is currently active. NCT04505189 from Imperial College is finished but not published. The third pilot study assessed the clinical tolerance and safety of 25mg psilocybin supported by psychological intervention in female AN patients with a BMI of 19 (Peck et al., 2023). The study found no significant changes in ECG, vital signs, or suicidality, and all adverse events (AEs) were mild and temporary. Two participants developed asymptomatic hypoglycemia post-treatment, which resolved within 24 hours with no other significant changes in laboratory values. Most participants reported subjectively meaningful experience, and a majority expressed that a second dose would have been beneficial. The study also observed significant reductions in eating disorder symptoms one-month post-treatment.

**Functional magnetic resonance imaging (fMRI) and psilocybin**

In a psilocybin fMRI study (Carhart-Harris et al., 2012), 30 volunteers underwent arterial spin labeling and BOLD fMRI assessments to observe the transition from a normal to a psychedelic state. The study revealed a noteworthy decrease in cerebral blood flow and BOLD signals, particularly in key brain regions such as the thalamus, anterior and posterior cingulate cortex. A significant finding was the reduced activity in the ACC/medial prefrontal cortex, correlating with the intensity of subjective psychedelic experiences.

In another study comparing psilocybin and escitalopram for major depressive disorder (Daws et al., 2022), psilocybin produced a rapid and lasting antidepressant response, linked to changes in brain network modularity measured by fMRI. This contrasted with the less pronounced antidepressant effect of escitalopram, which showed no significant changes in brain network organization.

In a recent longitudinal study comparing psilocybin and methylphenidate (Siegel et al., 2023, preprint), psilocybin showed a greater disruption in brain connectivity, particularly in the default mode network, involving desynchronization across spatial scales. Additionally, psilocybin induced a persistent decrease in functional connectivity between the anterior hippocampus and cortical regions related to the default mode network, with normalization observed after six months.

In summary, these studies collectively demonstrate that psilocybin induces profound alterations in brain function and connectivity. These alterations include decreased cerebral blood flow and activity in specific brain regions, changes in brain network organization, and disruptions in connectivity across various brain networks. These findings provide a deeper understanding of the neurobiological mechanisms underpinning the effects of psilocybin, with potential implications for its therapeutic use. However, research on the long-term effects of psychedelic compounds on neural processing is still limited and needs to be investigated further, specifically in relation to treatment effects in clinical trials (Wall et al., 2023).

**fMRI and AN**

Research on AN has produced a variety of findings regarding brain activation in patients (Fugleset et al., 2016). Studies highlight altered activation in fronto-striato and limbic circuits, pivotal in AN 's pathophysiology. Many studies have focused on limbic dysfunction, and its impact on emotional and perceptual neural circuits, using neural activation in relation to food cues, and body image distortion (i.e. disorder relevant stimulation protocols) as a model to investigate these issues (Bronleigh et al., 2022). These studies collectively indicate altered neural activity across multiple brain regions (frontal, parietal, temporal, occipital lobes, amygdala, striatum, thalamus, cerebellum), potentially contributing to various AN symptoms and behaviors.

One aspect of interest is how anxiety and pathological fear learning might lead to conditioned neural responses to food stimuli, potentially explaining the phobic avoidance of food in acute AN cases (Kaye et al., 2009). Limbic information processing in AN might be inhibited by cognitive areas like the dorsolateral prefrontal cortex and parietal cortex, possibly contributing to restrictive eating behaviors. While results from fMRI studies are diverse, they align interestingly with theories of neurocircuit dysfunctions in AN.

There's also evidence suggesting that increased activations in fronto-striatal circuits are linked to the maintenance of restrictive eating habits in AN (Steward et al., 2018). Functional network connectivity studies, though varied, generally indicate disrupted connectivity in executive networks, the default-mode network, and the salience network across eating disorders.

***Rationale for fMRI in a study of a psilocybin intervention for AN***

Functional MRI allows for the detailed observation of brain activity and connectivity changes as a function of treatment. Given that fMRI studies on psilocybin have revealed significant alterations in brain networks, particularly in regions associated with mood regulation and cognitive processing, it can provide critical insights into how psilocybin affects the brain regions implicated in AN. Moreover, fMRI has already demonstrated its utility in identifying altered neural activity in AN, particularly in circuits related to food perception, emotional processing, and body image. By using fMRI to track changes in these specific brain regions before and after psilocybin treatment, we can gain a deeper understanding of the neurobiological impact of psilocybin on AN and potentially identify biomarkers for treatment efficacy and optimization.

**Potential impact on synaptic plasticity**

Brain-Derived Neurotrophic Factor (BDNF) is a protein that plays a crucial role in neuroplasticity (Lohof et al., 1993; Cohen-Cory et al., 2010). Animal studies have shown that psilocybin promotes neurogenesis and synaptic plasticity (Moliner et al., 2023; Ly et al., 2018). It is indicated that 5-HT2A agonists increase cortical BDNF expression and BDNF protein levels in rodent brain neurons. Notably, individuals with AN exhibit reduced serum BDNF levels (Nakazato, 2003). However, the potential impact of psilocybin on BDNF levels in patients with AN remains unexplored. Investigating the relation between BDNF change before and after a two-dose psilocybin treatment in patients with AN could shed light on the neurobiological mechanisms of psilocybin’s action in the AN population and potentially provide valuable insight for treatment efficacy and optimisation.

3. Benefit-Risk Evaluation

Benefits:

Psilocybin, a naturally occurring psychedelic compound, has shown significant potential as a novel therapeutic intervention for various psychiatric conditions, particularly those characterized by treatment resistance and chronicity. Evidence from clinical trials has demonstrated significant antidepressant effects of psilocybin in populations with major depressive disorder (MDD) and treatment-resistant depression (TRD). In these trials, psilocybin has been associated with rapid and sustained reductions in depressive symptoms, with effects lasting weeks to months after a single or limited number of dosing sessions.

Beyond the reduction in symptoms, participants in these trials have often reported experiencing meaningful and transformative experiences, which they describe as personally insightful and emotionally significant. These self-reported experiences are believed to play a crucial role in the therapeutic effects of psilocybin, potentially leading to enhanced emotional processing, improved mood, and increased cognitive flexibility. These outcomes are particularly relevant for patients with anorexia nervosa (AN), a condition marked by rigid thought patterns, emotional avoidance, and a high degree of treatment resistance.

Currently, the available treatments for AN, including cognitive behavioural therapy (CBT) and pharmacotherapy, often result in limited efficacy and high relapse rates, particularly in cases where the disorder has become chronic. There is a critical need to explore new treatment modalities that can offer more substantial and sustained benefits. Psilocybin-assisted therapy represents a promising new avenue, especially considering its observed effects in enhancing cognitive flexibility and emotional insight, which are core areas of dysfunction in AN. Emerging research suggests that psilocybin-assisted therapy can address these gaps by fostering cognitive flexibility, reducing emotional rigidity, and enhancing the capacity for introspection and insight. This positions psilocybin as a potential new adjunctive intervention in the treatment of AN.

This study targets an age group (16-35 years) where AN often begins to develop into a chronic condition, making early intervention with innovative therapies particularly crucial. Individuals in this age range frequently show entrenched patterns of thought and behaviour that are resistant to standard treatments. By including this demographic, the study aims to evaluate whether psilocybin can disrupt these patterns, offering a potential shift in treatment outcomes that could prevent or mitigate the progression of AN into a more severe, chronic state.

In summary, the potential benefits of this study include:

- Evaluating the antidepressant and transformative effects of psilocybin in a population with few effective treatment options,
- understanding the role of meaningful self-reported experiences in therapeutic outcomes,
- Exploring potential biomarkers, such as BDNF levels and neuroimaging changes, to enhance future personalization of treatments.
- Targeting a critical age range for early intervention in anorexia nervosa.

**Risks and Adverse Events Associated with Psilocybin**

Previous research using doses of 30 and 40 mg reported no serious adverse events (Griffiths et al., 2006, 2011; Bogenschutz et al., 2022).

Key risks of psilocybin, as noted in literature, include prolonged psychotic symptoms, flashback phenomena or Hallucinogen Persisting Perceptual Disorder (HPPD), and anxiety reactions. Studies at the John Hopkins Center for Psychedelic Research, encompassing over 300 participants and 600 dosing sessions with 25mg psilocybin, recorded no cases of HPPD. HPPD itself is rare, with a prevalence of 4-4.5% among lifetime users of hallucinogens, though the specific incidence related to psilocybin is unclear (Kurtom et al., 2019). Prolonged psychotic responses in clinical psilocybin studies are infrequent, less than 1% (Studerus et al., 2011).

Anxiety during psilocybin experiences is common (Griffiths et al., 2006; Carhart-Harris et al., 2016a). A survey involving 1993 participants (mean age 29) exploring challenging experiences with psilocybin found that 84% benefited, despite psychological distress. The severity of the experience positively correlated with long-term increases in well-being (Carbonaro et al., 2016).

This study includes robust preparatory and integration sessions to minimize distress and maximize therapeutic outcomes. Therapists will receive specialized training to handle challenging experiences in real time.

Although the MRI examinations, during which patients need to lie still for about 1 hour, might be perceived as uncomfortable by some, they pose no health risk for the patient if routine safety precautions are followed (i.e. careful screening for contraindications for undergoing MRI and ensuring that no ferromagnetic metal objects are brought near the scanner).

**Suicidality**

A study on psilocybin for depression treatment noted two cases of suicidal behavior in the 25 mg group, compared to two in the 10 mg group and none in the 1 mg group within three weeks post-dosing (Goodwin et al., 2022). Another study comparing 25mg psilocybin with escitalopram for depression found no increase in serious adverse events or suicidal behavior in either group (Carhart-Harris et al., 2021).

This study includes active monitoring for suicidal ideation and behaviour during and after treatment sessions, with immediate intervention plans in place

**Specific clinical AN related risk**

One study of psilocybin 25mg for adults with AN, reported no clinically significant changes in ECG, vital signs, or suicidality levels (Peck et al., 2023). Only two participants experienced asymptomatic hypoglycemia post-treatment, which resolved within 24 hours. Additionally, no significant changes were noted in laboratory values. All adverse events (AEs) observed were mild and temporary. The overall participant feedback indicated that the treatment was generally well-received and deemed acceptable.

To mitigate potential risks, participants in this study will undergo comprehensive medical screening, including blood tests and ECG, before dosing session. A trained physician will be present during all psilocybin sessions to address medical emergencies, if needed.

4. Trial Objectives

For full list of measurements, please see attachments.

4.1 Primary Objective

- To assess the safety and tolerability of psilocybin 25 mg in adolescents and young adults with anorexia nervosa.

4.2 Secondary Objectives

- To evaluate the efficacy of psilocybin with psychological support in reducing AN symptom compared to TAU and time to relapse within 12-month follow-up.

- To investigate potential mechanisms of action through neuroimaging (fMRI), using both resting-state and task-based paradigms for estimation, and biomarker analysis (BDNF levels) with in six-month follow-up.

- To assess changes in mental health, well-being, and personality traits within 12-month follow-up.

- To conduct a qualitative analysis of the subjective experiences of patients, their relatives, and therapists at last follow-up at 12 months.

4.3 Primary Endpoint

- **Safety:** Incidence of adverse events (AEs) and serious adverse events (SAEs) during the trial period.

4.4 Secondary Endpoints

- Time to **Relapse:**

Defined as a composite outcome during the follow-up period, consisting of:

1. **≥7 % decrease in BMI** compared to post-hospitalization baseline BMI.

Rationale for 7 %: A 7% decrease in BMI reflects a significant deviation from expected weight gain in patients starting at a minimum BMI of 16, signaling potential relapse.

1. **Re-hospitalization for anorexia nervosa-related complications** (e.g., significant weight loss, medical instability). Data of hospitalization will be collected from hospital journal registers.
2. **Initiation of additional intensive psychiatric or nutritional interventions**, including day-patient or inpatient programs. Data of additional interventions will be collected from hospital journal registers.
3. **Self-reported significant deterioration in eating disorder symptoms** (e.g., severe food restriction, purging) as assessed by validated scales such as the Eating Disorder Examination Questionnaire 6.0 (EDE-Q 6.0).

**Rationale for Composite Outcome:**
This composite endpoint captures both clinical (BMI, hospitalization) and patient-centered (symptom deterioration) measures, aligning with the study’s aim to evaluate the efficacy of psilocybin in reducing the risk of relapse during a critical recovery period.

- Time to **Response:**

A clinically significant improvement in weight and psychological symptoms during the treatment period.

**Criteria**: **BMI Increase**: ≥10% increase in BMI from baseline (e.g., BMI 16 → BMI 17.6) or reaching a BMI ≥18.5 if achievable within the study period.

- Time to **Remission:**

The achievement and maintenance of weight restoration and psychological well-being, with no significant symptoms of anorexia nervosa.

**Criteria**: **Weight Restoration**: BMI ≥18.5 sustained for at least four weeks, or a weight within 90-95% of ideal body weight adjusted for age and height.

- Changes in AN symptom severity (measured by the Eating Disorder Examination from baseline to primary endpoint and follow-up 6 and 12 months.

- Changes in well-being from baseline to primary endpoint and follow-up 6 and 12 month.

- Changes in personality traits from baseline to primary endpoint and follow-up 6 and 12 months

- Changes in brain resting state connectivity (measured by fMRI), and commonly used task-based fMRI paradigms from baseline to primary endpoint. The task-based paradigms will involve food-related conditions, commonly used in the population (Celeghin et al., 2023; Bronleigh et al., 2022) as well as established paradigms involved in processing reward anticipation (Knutson et al., 2000; Ventorp et al 2022)

- Changes in peripheral BDNF measured in platelet-poor EDTA plasma from baseline to primary endpoint.

5. Trial Design and Procedures

5.1 Overall Trial Design

- A Phase IIa, open-label, randomized controlled trial with two arms:

1. Active treatment arm; Two dosing sessions with psilocybin 25mg with psychological support alongside TAU.

2. Active comparator control arm; TAU only.

TAU includes specialized care offered in an eating disorder unit in Region Skåne, Sweden. If the active treatment arm is determined to be safe, tolerable, and preliminarily effective during the follow-up assessment, participants in the control group will have the option to switch to the active treatment while maintaining their usual specialized care.

This design minimizes ethical concerns regarding withholding a potentially effective treatment.

Participants will be randomly assigned (1:1) to either the intervention or control group.

Block randomization stratified by age group (16–17 and 18–35 years) will ensure balanced representation.

**Rationale for fixed psilocybin 25mg dosing:**

The selected fixed dose of 25 mg is based on extensive use in prior clinical trials, including COMP005 and COMP006 (phase III), and the COMP007 long-term safety follow-up study. All of these protocols employed a fixed 25 mg dose without weight-adjustment and were approved by regulatory agencies in multiple countries. These trials have consistently demonstrated that 25 mg is safe, well-tolerated, and associated with meaningful therapeutic effects.

This approach is also supported by prior studies involving patients with anorexia nervosa, which used similar inclusion criteria and demonstrated safety and therapeutic benefit of the 25 mg dose (Peck et al., 2023). Furthermore, as noted by MacCallum et al. (2022), a secondary analysis of prior psilocybin trials found no significant difference in psychedelic effects between fixed 25 mg dosing and weight-adjusted doses (e.g., 0.29–0.43 mg/kg), reinforcing the rationale for a standardized dose regardless of body weight.

**Rationale for dosing interval:**

Several clinical trials have consistently demonstrated that administering two doses of psilocybin spaced 2 to 4 weeks apart is both safe and therapeutically appropriate when delivered under professional supervision with structured integration support (Bogenschutz et al., 2022; Carhart-Harris et al., 2021; Davis et al., 2021; Garcia-Romeu et al., 2015; Gukasyan et al., 2022). These dosing intervals are grounded in empirical findings from multiple populations and indications. For instance, Carhart-Harris et al. (2021) administered two 25 mg doses of psilocybin three weeks apart to 59 individuals with depression, reporting no serious adverse events (SAEs) and only transient effects such as headache and nausea. Garcia-Romeu et al. (2015) conducted a feasibility study in 15 smokers, administering 20 mg/70 kg and 30 mg/70 kg doses spaced by approximately two weeks; again, no SAEs were reported, and the design highlighted psychological integration. Davis et al. (2021) employed a two-week interval between doses in 24 patients with depression. Finally, Bogenschutz et al. (2022) studied 93 individuals with alcohol use disorder, spacing two 25 mg doses four weeks apart, with no serious adverse drug reactions noted. Collectively, these studies support the 4 week interval as an evidence-based standard that balances therapeutic efficacy with participant safety.

**Rationale for open label randomized controlled trial (not blinded)**

While blinding increases scientific rigor, the likelihood of functional unblinding is high due to the strong experiential contrast between 25 mg psilocybin and a sub-threshold dose such as 1 mg or placebo. This substantially limits the scientific value of using 1 mg psilocybin as an active control. Notably, published trials using low-dose or placebo comparators have not assessed blinding effectiveness—likely because participants can easily distinguish active treatment. These studies should therefore be considered functionally unblinded.

We do not consider it ethical to simulate a blinded design simply for its perceived rigor, when functional unblinding undermines its validity. According to our experience from other studies, participants receiving 1 mg frequently reported frustration at having to undergo 5–6 hours of intensive procedures without any noticeable effects. This is experienced as “placebo,” may lead to reduced trust in the study.

These concerns are amplified in individuals with anorexia nervosa—a psychologically and medically vulnerable group. Extended placebo procedures (e.g., preparation, fasting, lying still with eye mask and music) may cause unnecessary psychological distress when the experience is perceived as ”empty”. Such reactions can negatively affect motivation, retention, and therapeutic alliance.

**Rationale for concomitant antidepressant treatment**

The primary pharmacodynamic mechanism of psilocybin is mediated through agonism at the 5-HT2A receptor. Consequently, medications with direct 5-HT2A-receptor antagonistic effects may block or substantially attenuate the effects of psilocybin and are therefore excluded from psilocybin administration.

In contrast, commonly prescribed antidepressants such as selective serotonin reuptake inhibitors (SSRIs) and serotonin–norepinephrine reuptake inhibitors (SNRIs) do not exert direct 5-HT2A-receptor antagonism. Mandatory discontinuation of these medications, in the absence of a clear mechanistic or safety-based rationale, may expose participants to unnecessary clinical risk, including symptom deterioration and reduced treatment stability.

Given that the present study targets individuals with anorexia nervosa rather than depressive disorders, antidepressant efficacy per se is not a primary outcome. Requiring discontinuation of antidepressants solely based on practices derived from depression trials would therefore be disproportionate in this context.

Available controlled human data indicate that concomitant treatment with SSRIs does not necessarily attenuate the subjective or physiological effects of psilocybin and may reduce anxiety-related adverse effects (Becker et al., 2025). Furthermore, antidepressant discontinuation is associated with clinically relevant risks, including withdrawal symptoms and symptom destabilization. At the same time, observational data suggest that ongoing or recent SSRI/SNRI use may attenuate certain subjective effects of psilocybin, with some reports indicating that this attenuation could persist for several weeks or even months following discontinuation (Gukasyan et al., 2023). However, the magnitude, consistency, and clinical relevance of this potential reduction remain uncertain.

Allowing continued antidepressant treatment when clinically indicated improves patient safety, reduces avoidable treatment disruption, and increases the representativeness of the study population relative to real-world clinical care. This approach supports proportionality and ethical conduct while maintaining the scientific integrity of the intervention and its mechanistic rationale.

**Impact on Treatment as Usual (TAU)**

In this study, the control group receives Treatment As Usual (TAU), consisting of evidence-based care within specialized eating disorder services. Introducing a “placebo” experience with 1 mg psilocybin—when participants recognize it as inactive—may undermine their engagement with TAU. This could introduce a negative expectancy bias, leading to demoralization or nocebo-like effects that reduce adherence and treatment response. In effect, the placebo condition could contaminate the comparator and bias outcomes against the control group, compromising both scientific validity and ethical responsibility.

**Study Design Justification and Endpoint Suitability**

This trial is designed to assess safety and tolerability in a vulnerable population. Results will guide future trials where efficacy becomes the primary objective. At that stage, a larger sample and more complex control design may be justified.

Our current outcome measures—such as BMI change, re-hospitalization, and BDNF—are relatively objective and well-suited for detecting clinically meaningful effects, even without a placebo comparator.

**Statistical Consideration**

With only 40 participants, statistical power to detect between-group differences is limited regardless of blinding. Thus, the trial is appropriately focused on safety, feasibility, and tolerability. Qualitative and neurobiological measures are included to support interpretation.

**Scientific Precedent and Stepwise Development**

As described in the protocol, this trial aligns with established guidance for early-phase psychedelic research (e.g., Johnson et al., 2008). Open-label designs have been widely accepted in early trials involving vulnerable populations and novel treatments. This stepwise approach provides critical groundwork for future efficacy-focused studies.

**Clarification on Targeted Sample Size for Dosing**

The trial aims to dose at least 20 participants in the psilocybin treatment arm. This number refers to participants who receive at least one 25 mg psilocybin session, not only those who are randomized.

Analysis of safety and tolerability will primarily focus on this dosed cohort.

Recruitment will continue until a minimum of 20 participants in the intervention arm have received at least one dose, unless safety or feasibility concerns require early termination.

**5.2 Procedures and Schedule of Assessment**

All procedures will be conducted at the University Hospital for Psychiatry, Baravägen 1, Lund, except the fMRI assessments which are performed at the The National 7T Facility in Lund. All assessments will be carried out by qualified personnel appointed by the principal investigator, including medical doctors, nurses, and psychologists. The National 7T Facility will appoint qualified personnel for fMRI assessment.

The duration of the entire trial is from the first screening of the first patient to the last follow up of the last patient. For each patient participant, the duration of the trial is from the screening to the last follow up at week 52 (12 month).

Patient rehospitalization and additional interventions data are collected in patient journal registers.

**Pre-Study Activity**

Following ethical approval, a focus group will be conducted with patients with anorexia nervosa in two different groups, one aged 16–18 and one 19–35 years. The purpose is to provide study information, gather feedback on the clarity and ethical aspects of the protocol, and identify ways to improve potential benefit. Input from this focus group will inform study quality, recruitment materials, communication strategies and ethical aspects of psilocybin research experienced by the population. Any amendments based on this will be processed according to CTIS protocol.

**Screening Phase**

A brief telephone pre-screening is performed before the screening visit to avoid unnecessary visits for patients. This is conducted following verbal permission to access medical records and a verbal consent to a short pre-screening assessment to determine whether participants may qualify for full screening. Screening includes psychiatric and medical history, inclusion/exclusion criteria assessment, safety blood tests (glucose, liver, kidney), and, when clinically indicated additional laboratory tests to exclude common medical conditions and support eligibility assessment, ECG, informed consent (with a 2-week consideration period), pregnancy test and urine toxicology (U-tox). The time from screening to the first psilocybin dosing session must not exceed 8 weeks.

**Preparation Phase**

- Preparation Session 1 & Baseline Assessment (Week 0): Psychoeducation about psilocybin, breathing and relaxation techniques, rapport-building with therapists, and discussion of expectations and concerns.
  Includes full baseline assessments (list provided as attachment to protocol:
  - Expectation of Treatment Scale (ETS-BF)
  - Readiness and Motivation Questionnaire (RMQ)
  - General Change Mechanisms Questionnaire (GCMQ)
  - Patient Health Questionnaire (PHQ-9)
  - Generalized Anxiety Disorder scale (GAD-7)
  - Eating Disorder Examination Questionnaire (EDE-Q )
  - Columbia-Suicide Severity Rating Scale (C-SSRS)
  - Brief Psychiatric Rating Scale – Extended (BPRS+)
  - Satisfaction With Life Scale (SWLS)
  - Positive and Negative Affect Schedule (PANAS)
  - Harmony in Life Scale (HILS)
  - Ten Item Personality Inventory (TIPI)
  - Honesty-Humility Scale (HH)EGON
- Visual size estimation (EGON)

Vital signs, ECG, blood glucose, urine drug screening, pregnancy test, fMRI, BMI and blood sampling for BDNF and safety labs are also conducted.

Preparation Session 2 (Week 1): Conducted 7–10 days after Preparation 1, and 2–3 days before psilocybin dosing.

**Dosing and Integration Phase**

Dosing Session 1 (Week 1): Psilocybin 25 mg under therapeutic support with ECG, blood glucose and blood pressure/pulse monitoring.

Integration Session 1 (Day after Dosing 1): Reflection, fMRI, blood sampling (including glucose, liver, kidney, BDNF), and reassessments with RMQ, GCMQ, PHQ-9, GAD-7, C-SSRS, BPRS+, SWLS, PANAS, HILS, TIPI, ECG and blood pressure/pulse. Psychedelic Experience related scales; Altered States of Consciousness Rating Scale (5D-ASC), Mystical Experience Questionnaire (MEQ-4), Meaningful Life Experience Rating (MLE).

Integration Session 2 (Week 2): Continued psychological integration support.

Integration Session 3 (Week 3): Summary of first dosing experience and preparation for second dosing. A pregnancy test is performed.

Dosing Session 2 (Week 4): Second psilocybin 25 mg administration under identical conditions as first dosing session.

Integration Session 4 (Day after Dosing 2): Reflection, blood sampling (including glucose, liver, kidney, BDNF), and reassessments with RMQ, GCMQ, PHQ-9, GAD-7, C-SSRS, BPRS+, SWLS, PANAS, HILS, TIPI, ECG and blood pressure/pulse. Psychedelic Experience related scales; Altered States of Consciousness Rating Scale (5D-ASC), Mystical Experience Questionnaire (MEQ-4), Meaningful Life Experience Rating (MLE).

Integration Session 5 (Week 5): Final integration session and preparation for long-term follow-up.

**Primary Endpoint Week 9**

Includes full safety and outcome evaluations:
- fMRI

- blood sampling (including glucose, liver, kidney, glucose, BDNF)
- Vital signs, ECG, U-tox

- BMI
- AE/SAE monitoring
- Readiness and Motivation Questionnaire (RMQ)
- General Change Mechanisms Questionnaire (GCMQ)
- Patient Health Questionnaire (PHQ-9)
- Generalized Anxiety Disorder scale (GAD-7)
- Eating Disorder Examination Questionnaire (EDE-Q)
- Columbia-Suicide Severity Rating Scale (C-SSRS)
- Brief Psychiatric Rating Scale – Extended (BPRS+)
- Satisfaction With Life Scale (SWLS)
- Positive and Negative Affect Schedule (PANAS)
- Harmony in Life Scale (HILS)
- Ten Item Personality Inventory (TIPI)
- Honesty-Humility Scale (HH)

- Visual size estimation (EGON)

**Intensive Follow-Up Phase Month 3,4 and 5**

Follow-up visits include:
- Eating Disorder Examination Questionnaire (EDE-Q)
- Columbia-Suicide Severity Rating Scale (C-SSRS)
- Brief Psychiatric Rating Scale – Extended (BPRS+)
- AE/SAE monitoring
- BMI, blood pressure, ECG
- Glucose, U-tox

**6-Month Follow-Up**

Same as primary endpoint assessments (except not fMRI), including:

- blood sampling (including glucose, liver, kidney, BDNF)
- Vital signs, ECG, U-tox

- BMI
- AE/SAE monitoring
- Readiness and Motivation Questionnaire (RMQ)
- General Change Mechanisms Questionnaire (GCMQ)
- Patient Health Questionnaire (PHQ-9)
- Generalized Anxiety Disorder scale (GAD-7)
- Eating Disorder Examination Questionnaire (EDE-Q )
- Columbia-Suicide Severity Rating Scale (C-SSRS)
- Brief Psychiatric Rating Scale – Extended (BPRS+)
- Satisfaction With Life Scale (SWLS)
- Positive and Negative Affect Schedule (PANAS)
- Harmony in Life Scale (HILS)
- Ten Item Personality Inventory (TIPI)
- Honesty-Humility Scale (HH)

- Visual size estimation (EGON)

**Extended Follow-Up Phase**

9-Month Follow-Up

- Eating Disorder Examination Questionnaire (EDE-Q)
- Columbia-Suicide Severity Rating Scale (C-SSRS)
- Brief Psychiatric Rating Scale – Extended (BPRS+)
- AE/SAE monitoring
- BMI, blood pressure, ECG
- glucose, U-tox

12-Month Final Follow-Up
- Eating Disorder Examination Questionnaire (EDE-Q)
- Columbia-Suicide Severity Rating Scale (C-SSRS)
- Brief Psychiatric Rating Scale – Extended (BPRS+)

- Patient Health Questionnaire (PHQ-9)
- Generalized Anxiety Disorder scale (GAD-7)

- Ten Item Personality Inventory (TIPI)

- Meaningful Life Experience Rating (MLE).
- AE/SAE monitoring
- BMI, blood pressure, ECG
- glucose, U-tox

Participants who show signs of psychological or physical deterioration at any point during the study between follow-ups are instructed to contact the research team at any time and will be offered additional assessment and support.

***Description of Psilocybin Administration and Psychological support***

Psychological support includes a non-directive preparation and integration pre- and post-dosing sessions according to protocol manual, alongside support for the patient on the dosing session day. The study follows the guidelines for safe research with psychedelics (Johnson et al, 2008).

Preparation session

Standard preparation in psilocybin clinical trials including psychoeducation of the effects of psilocybin, breathing techniques, getting to know the two therapists (at least one licensed health care professionals experienced with eating disorders and trained in psilocybin therapy including either a psychologists, psychiatrists, nurse and physiotherapist, ) , one male and one female), the onsite psychiatrist on call during dosing session, and other relevant professionals of the research team, practicing how to manage challenging episodes of the dosing session, agreeing on contracts for touch and being able to ask questions. A standardized preparation script will ensure consistency across participants. The assistant therapist can be non-licensed healthcare personnel experienced with the anorexia nervosa population, such as a healthcare assistant. All psychological support therapists must have done all specific 5-day training in the psiAN manual.

Dosing session day

The dosing session, lasting 6-8 hours, is supported by the lead and assisting therapists introduced during preparation sessions. The psilocybin's acute effects persist for 4-6 hours, recorded via video and audio. Participants, lie down with an optional eye mask, experience the session in a comfortable room with a pre-selected music playlist, respecting individual preferences.

Therapists provide support and guidance if requested but with minimal psychotherapeutic focus.

Therapists will follow pre-established protocols for de-escalation and grounding in case of distressing experiences.

Parents are introduced at the session's end with participant approval.

During the dosing session, a medically trained study doctor will be available, equipped for emergencies in the unlikely event of serious adverse events related to psilocybin risks.

Integration session

The participant will be back at the research facility two times after each dosing session for integration sessions (approx. 1-2 hours) with the therapists to further explore the dosing session and its effects.

Integration sessions will include structured discussions about insights gained, with therapists facilitating connections between the experience and the participant’s therapeutic goals.

**Monitoring Post-Psilocybin Administration adverse events between visits.**

Participants who experience significant psychological distress, abnormal vital signs, or other health concerns between study visits are instructed to contact the research team during daytime hours using the contact details provided in the participant information sheet. During evenings or weekends, participants should seek assistance from local emergency services as appropriate.

5.3 Biological Sampling Procedures

Blood samples (up to 5 ml per participant per occasion) will be collected for the analysis of Brain-Derived Neurotrophic Factor (BDNF) as an exploratory biomarker and for tolerance and safety measurements. Additionally, we aim to collect one tube (5 ml = 10ml in total) of additional whole blood per occasion for future analysis.

BDNF samples will be taken at five key time points: (1) before treatment (baseline), (2) and (3) at first integration session after Psilocybin 25mg dosing, (4) at 5 weeks, and (5) during the 6-month follow-up. This ensures comprehensive longitudinal data collection.

Blood samples of glucose, kidney and liver status will be measured for safety and tolerance reasons. None of these blood samples are collected or stored.

All blood samples are done by a standard peripheral venous sampling method performed by a nurse at the research facility at the university hospital clinic for psychiatry at Baravägen 1, Lund.

Procedures will be implemented to minimize discomfort during blood collection, such as using pediatric needles for younger participants when necessary. Blood collection and processing will follow standardized protocols to ensure sample integrity.

Handling, Storage, and Destruction of Biological Samples:

Blood samples taken for analysis of BDNF will be analyzed at the Wallenberg Neuroscience Center or Avdelningen för neurokemi, thereafter stored at Region Skåne Bio bank for maximum 10 years under secure conditions and in accordance with Swedish regulations and the protocol for “Följsamhet med regler för hantering av biologiska prov”.

5.4 Start, End, Temporary Halt, and Early Termination

- Start of the Clinical Trial: Defined as the first visit of the first participant (screening visit).

- Temporary Halt or Early Termination: Will occur if significant concerns arise from the sponsor, monitoring oversight or Safety Review Committee (see 9.1).

***Halt and/or Early Termination Criteria***

Halt and/or Early termination may be initiated if any of the following occurs:

- Unacceptable or unforeseen safety concerns, including unexpected serious adverse reactions (SUSARs) or a pattern of adverse events suggesting increased risk.
- Insufficient recruitment, defined as fewer than 30% of the planned number of participants being enrolled within 18 months of trial initiation.
- Emerging external evidence (e.g. from other clinical trials or meta-analyses) indicating that the investigational product poses a significant safety risk or is no longer ethically justifiable to study.
- Ethical concerns raised by the Safety Review Committee, monitor oversight or regulatory authorities.
- Withdrawal of essential approvals, resources, or study drug availability.

In the event of early termination or temporary halt, all relevant regulatory authorities (including CTIS, Läkemedelsverket, and EPN) will be notified within 15 calendar days, as per applicable regulations. The reason for halt and/or early termination will be documented and explained in the final report.

Safety Review committee and monitor clearance will be required before resuming after a temporary halt not leading to and early termination.

- End of the Clinical Trial: When the last participant completes the last follow-up visit at week 52.

6. Subject Selection

6.1 Inclusion Criteria

- Diagnosis of AN per DSM-5, including AN in partial remission.

- Have experienced at least one period of weight restoration to a minimum BMI of 17 followed by subsequent weight loss.

- Age 16-35.

- BMI >16

- Stable contact with a psychiatric unit.

- Ability to provide informed consent.

6.2 Exclusion Criteria

- Psychosis, bipolar disorder, substance use disorder, family history of psychosis or bipolar disorder, refusal of birth control, lifetime psychedelic use.
- Cardiovascular conditions,
  - Resting systolic blood pressure >140 mmHg or diastolic blood pressure >90 mmHg at screening or baseline.
  - Clinically significant arrhythmias, tachycardia and QT prolongation.
  - History of stroke, myocardial infarction, or other significant cardiovascular events.
  - Seizure disorders or history of epilepsy.
- Diabetes mellitus, positive drug tests, suicidal intent, allergy or intolerance to drug content, blood or needle phobia.
- Metal pieces in the body (contraindicated by fMRI, assessed in each case by MR-technician)
  - Only for the MRI-part of the study
- Any other clinically significant medical condition that, in the investigator’s opinion, may pose a risk to the participant or interfere with study results.
- Care under the Swedish Compulsory Psychiatric Care Act (LPT).
- Ongoing treatment with medications that have clinically relevant 5-HT2A-receptor antagonistic properties and are therefore expected to directly block or substantially interfere with the pharmacodynamic effects of psilocybin. Such medications require an appropriate washout period prior to psilocybin administration, based on pharmacokinetic properties and clinical judgement. Medications without direct 5-HT2A-antagonistic effects are not excluded on this basis**.**
- Sustained weight restoration, defined as BMI > 21.0 for ≥ 6 consecutive months within the 12 months prior to screening, as documented in medical records.

6.3 Screening and Inclusion

- Potential participants will be screened by a psychiatric clinician appointed by the principal investigator to ensure eligibility and understanding of the study requirements. A brief telephone pre-screening is performed before the screening visit to avoid unnecessary visits for patients. This is conducted following verbal permission to access medical records and consent to a short pre-screening assessment. Screening blood tests may be performed to exclude common medical conditions and support eligibility assessment.

6.4 Withdrawal and Discontinuation Criteria

**Discontinuation from the Clinical Trial**

A participant will be discontinued entirely from the clinical trial (i.e., all further participation and follow-up will end) only under the following condition:

- Withdrawal of informed consent at any time, for any reason, without the need to justify.

**Discontinuation from the Intervention (Dosing)**

Participants may be discontinued from the intervention (i.e., psilocybin administration – first or second dose), without being excluded from the trial. Participants will be encouraged to continue with follow-up assessments unless they explicitly withdraw consent.

This approach allows for continued safety and data collection in accordance with the intention-to-treat principle.

Reasons for discontinuing intervention may include:

- Development or discovery of exclusion criteria after inclusion (e.g. new psychiatric diagnosis, pregnancy).
- Emergence of a serious adverse event (SAE) or medical condition that, in the investigator’s judgment, makes continued treatment unsafe.
- Initiation of treatment with medications prohibited according to the protocol, i.e. medications with clinically relevant 5-HT2A-receptor antagonistic properties.
- Failure to adhere to critical aspects of the study protocol (e.g. repeated missed visits, non-compliance with preparation, or safety procedures).
- Investigator decision in consultation with the medical monitor.

The reason for discontinuation is documented in the eCRF. Participants will be offered a final follow-up visit when appropriate.

- Non-compliance to fMRI will not lead to study exclusion nor discontinuation of the intervention.

7. Trial Treatments

7.1 Description of Investigational Medicinal Products

Psilocybin: 25 mg, administered orally in a controlled clinical setting with manual based psychological support. The time for dosing is 10am and the duration of the dosing session is approximately 6-8 hours.

**Packaging of Investigational Medicinal Product**

- The investigational medicinal product PEX010 (psilocybin 25 mg capsules) is manufactured and packaged by Psilo Scientific Ltd., Burnaby, BC, Canada.
- The product is packaged in high-density polyethylene bottles with silica desiccant and cotton to ensure stability and protection from moisture.
- Each bottle contains the required number of capsules per study protocol and is sealed with induction technology to prevent contamination.

**Labelling of the IMP**

- Labelling of the investigational medicinal product and auxiliary medicinal products will comply with the EU Clinical Trials Regulation (CTR No. 536/2014), Chapter X, Articles 66-67, Annex VI, Sections A to D. All text on label is in English.
- The label will include, at a minimum:
  - Study title and protocol number
  - Unique batch and lot number
  - Expiry date or retest date
  - Storage conditions
  - Statement indicating “For clinical trial use only”
  - Contact information for the sponsor
  - The required regulatory caution statements as per EU CTR and Swedish regulatory guidelines
- A sample of the label will be provided as an appendix to the clinical trial application.

**Storage and Handling of the IMP**

- The PEX010 psilocybin capsules must be stored in a temperature-controlled environment at 15-30°C, as specified in the Certificate of Analysis (COA)​.
- The product must be protected from direct light and moisture to maintain stability and integrity​.
- Storage conditions at each clinical site must meet GMP and GDP (Good Distribution Practice) standards, and IMP storage will be regularly monitored to ensure compliance.

**Transport and Distribution**

- The IMP will be imported by Oriola and distributed in compliance with EU GDP regulations.
- During transportation, the IMP must be maintained at the required 15-30°C temperature range using validated packaging solutions.

**Accountability and Documentation**

- Site personnel will document all IMP storage, dispensing, and destruction in an IMP accountability log.
- The investigational product will be dispensed only to authorized personnel and must be administered strictly per protocol.
- IMP reconciliation will be conducted at study closeout, and unused product will be returned or destroyed in accordance with regulatory requirements.

7.2 Auxiliary Medicinal Products

- None planned.

- In cases of significant distress or adverse reactions during dosing sessions, adjunctive medications such as benzodiazepines (e.g., lorazepam) may be administered at the discretion of the study physician. These instances will be documented and reported to monitoring oversight and safety monitoring committee.

7.3 Concomitant Use of Other Medicinal Products and Treatments

Concomitant use of psychiatric medications may be permitted if not listed below and if deemed safe by the principal investigator.

**Participants receiving ongoing treatment with antipsychotic medications or lithium will generally not meet the inclusion criteria for this study, as such treatments typically indicate psychiatric conditions that are exclusionary according to the protocol and/or involve pharmacological mechanisms that are incompatible with psilocybin administration.**

**In cases where the underlying diagnosis does not in itself constitute an exclusion criterion, and a participant is receiving a medication with clinically relevant 5-HT2A-receptor antagonistic properties off-label or for other non-exclusionary indications, the investigator may, together with the participant, carefully assess the feasibility of dose reduction or discontinuation. If dose reduction or discontinuation is not clinically appropriate or feasible, the participant will not be included in the study.**

**Tricyclic antidepressants (TCAs) exhibit clinically relevant 5-HT2A-receptor antagonistic properties. Ongoing treatment with TCAs is rare in the target population. In the rare event of TCA treatment, careful clinical assessment must be undertaken to evaluate the medical necessity and feasibility of discontinuation. If dose reduction or discontinuation is not clinically appropriate or feasible, the participant will not be included in the study.**

**Mirtazapine, which has pronounced 5-HT2A-receptor antagonistic effects, may be considered for gradual tapering and discontinuation prior to psilocybin administration. If dose reduction or discontinuation is not clinically appropriate or feasible, the participant will not be included in the study.**

**Antihistamines with 5-HT2A-receptor antagonistic properties will be assessed on a case-by-case basis. As these medications are typically prescribed on an as-needed basis, temporary discontinuation prior to dosing is generally feasible.**

**Selective serotonin reuptake inhibitors (SSRIs) and serotonin–norepinephrine reuptake inhibitors (SNRIs) do not exhibit direct 5-HT2A-receptor antagonism and are therefore not prohibited per se.**

**All concomitant medications will be carefully documented and considered in the interpretation of clinical outcomes.**

The following psychiatric medications are prohibited in participants receiving psilocybin due to known or potential pharmacodynamic interactions and risk of attenuated psychedelic effects or adverse reactions:

•Monoamine oxidase inhibitors (MAOIs)

•Tricyclic antidepressants (TCAs)

•Benzodiazepines ≤ 2 mg lorazepam equivalents per day (must not be taken within 12 hours prior to psilocybin administration)

•Antipsychotics

•Lithium

Participants will undergo a thorough medication review during screening to identify potential pharmacological interactions. Medications with prohibited serotonergic activity require washout periods of 2–4 weeks before psilocybin dosing according to clinical practise guidelines.

- Participants who cannot safely discontinue contraindicated medications will not be included in the study.

- The washout will primarily be monitored by phone and a medical doctor within the research team appointed by the principal investigator, with in-person visits if needed. Participants who exceed the 8-week limit without dosing will be re-evaluated for eligibility by the principal investigator. The time from screening to first dosing shall not exceed 8 weeks regardless of washout status.

- Psychotherapy and other non-pharmacological treatments will be allowed throughout the study period as TAU if not deemed an exclusion criterion. Temporary daycare after discharge will be considered part of TAU rather than non-pharmacological treatment.

7.4 Treatment After Trial End

- Participants will return to standard clinical care after the trial concludes. No continuation of psilocybin treatment is planned.

A transition plan will ensure continuity of care, including referrals to specialists or therapists for participants requiring additional support.

After the follow-up at six months, and once the safety committee has determined that safety and preliminary efficacy are satisfactory, participants in the active comparator arm will be offered the option to switch to the psilocybin intervention arm.

The switch to psilocybin treatment will follow the same preparation, dosing, and integration protocols as outlined for the intervention group.

8. Methods for Measurement of Endpoints for Clinical Efficacy and Safety

See attachment for complete list of measurement.

8.1 Methods for Measurement of Endpoints for Clinical Efficacy

Composite Relapse Endpoint:

- - BMI Decrease: Measured at baseline, 5 weeks, and 6 months using calibrated equipment and standardized protocols.
  - Hospitalization Data: Collected through patient reports and confirmed by medical records.
  - Symptom Deterioration: Assessed using validated tools such as the EDE-Q 6.0 and clinical interviews conducted by trained staff.
  - Clinical Intervention Use: Recorded in patient files, including initiation of new treatments during follow-up.

Changes in AN symptom (EDE-Q 6.0).

8.2 Methods for Measurement of Endpoints for Clinical Safety

Continuous clinical safety monitoring will be performed by licensed healthcare professionals at Lund University Hospital throughout the trial, from baseline to the final 12-month follow-up. The safety evaluations cover physical, biochemical, and psychological parameters relevant to psilocybin administration.

For a complete list and overview, please visit the Schedule of Assessments attached to the protocol.

- **Hepatic and renal function:** Blood samples will be collected to assess creatinine, albumin, PK-INR, ALAT, ASAT, GT, ALP, and bilirubin levels. These assessments will occur during screening, at baseline (Week 0), after each psilocybin dosing, at the primary endpoint, and at the 6-month follow-up (Week 24).
- **Glucose:**Measured at the same time points as above – during screening, baseline, after each dosing, at the primary endpoint, and the 6-month follow-up. Additionally, glucose levels will be assessed at 9- and 12-month follow-up visits.
- **Urine toxicology (U-tox):** Drug use screening will be performed at baseline, and again at 6-, 9-, and 12-month follow-ups.
- **Cardiovascular parameters (ECG, blood pressure, pulse):** These will be measured at screening, baseline, after each dosing session, during the first integration sessions (1 day post-dosing), and at all subsequent follow-ups.
- **Assessment of suicidality and psychiatric safety:** Suicidality will be assessed using the Columbia-Suicide Severity Rating Scale (C-SSRS) at baseline, after each psilocybin session, during integration, and at all follow-ups. Psychiatric symptoms will be monitored using the Brief Psychiatric Rating Scale – Extended (BPRS+).
- **Mental health symptoms:** Self-report instruments including the PHQ-9 (depression), GAD-7 (anxiety), and other measures will be completed at baseline, at each integration visit, at the primary endpoint, and at the 12-month follow-up.
- **Adverse Events (AE/SAE):** Spontaneously reported by participant patients or by clinician at all in-person visits from first dosing onwards, including all integration and follow-up assessments.

All data will be collected under Good Clinical Practice (GCP) conditions and entered the electronic case report form (eCRF) by trained personnel.

9. Handling of Adverse Events

9.1 Definitions

- *Adverse Event (AE):* Any untoward medical occurrence in a subject to whom a medicinal product is administered and which does not necessarily have a causal relationship with this treatment.

*Adverse Reaction (AR):* In the pre-approval clinical experience with a new medicinal product or new use of a medicinal product, and particularly as the therapeutic dose(s) may not be established, all noxious and unintended reactions to the medicinal product related to any dose should be considered an adverse reaction (AR). The phrase “reaction” to a medicinal product means that the causal relationship between the medical product and an adverse event is at least a reasonable possibility, that is the relationship cannot be ruled out.

*Serious Adverse Event (SAE) Serious Adverse Event (SAE): Any untoward medical occurrence that at any dose requires inpatient hospitalization or prolongation of existing hospitalisation, results in persistent or significant disability or incapacity, results in a congenital anomaly or birth defect, is life-threatening, or results in death.*

*Medical and scientific assessment will be made to determine if an event is serious.*

*Suspected Unexpected Serious Adverse Reactions (SUSAR)*: An adverse reaction/event that is unexpected, serious, and suspected to be caused by the treatment, i.e. adverse reactions/events that are not included in the RSI section of the Investigator’s Brochure (IB) or SmPC.

9.2 Assessment of Adverse Events

-Events will be assessed by the clinical team for causality, intensity, and seriousness and potential relationship to treatment (psilocybin 25mg).

The investigator is responsible for determining whether there is a causal relationship between the AE/SAE and use of the investigational medicinal product.

Consideration should be given to whether there is a reasonable possibility of establishing a causal relationship between the adverse event and the investigational medicinal product based on the analysis of the available evidence.

All AE can be categorized as either likely related, possibly related, unlikely related or not related, in accordance with the definitions below:

Likely related: Clinical event, including abnormal results from laboratory analyses, occurring within a reasonable time after administration of the intervention/investigational medicinal product. It is unlikely that the event can be attributed to underlying disease or other medications but is most likely caused by the investigational medicinal product and its emergence is reasonable in relationship with use of the investigational medicinal product.

Possibly related: Clinical event, including abnormal results from laboratory analyses, occurring within a reasonable time after administration of the intervention/investigational medicinal product. The event could be explained by the investigational medicinal product and its emergence is reasonable in relationship with use of the investigational medicinal product, but there is insufficient information to determine the relationship. The event could be explained by an underlying disease or other medications.

Unlikely related: Clinical event, including abnormal responses from laboratory tests, unlikely to be related to the intervention/investigational medicinal product and can be reasonably explained by other medication or underlying disease.

Not related: Clinical event, including abnormal results from laboratory analyses, that is not reasonably related to the use of the intervention/investigational medicinal product.

Those AEs which are suspected of having a causal relationship to the investigational medicinal product will be followed up until the subject has recovered or is well taken care of and on the way to good recovery.

If the reporting investigator does not provide any information on causality, the sponsor should consult with the reporting investigator and encourage the expression of a position on this issue. The sponsor must take into account the assessment of causality provided by the investigator. If the sponsor disagrees with the investigator's assessment of causality, both the investigator's and the sponsor's views should be included in the report.

Each adverse event shall be classified by an investigator as mild, moderate or severe.

Mild: The adverse event is relatively tolerable and transient in its nature but does not affect the subject’s normal life.

Moderate: The adverse event causes deterioration of function but does not affect health. The event can be sufficiently unpleasant and interferes with normal activities but does not completely obstruct them.

Severe: The adverse event causes deterioration of function or work ability or poses a health risk to the subject.

9.3 Reporting and Registration of Adverse Events

- AEs will be recorded by clinicians in the research team at each contact point with a study participant and reported in the REDCap documentation system and a manual standardized AE log template and serious events will be reported to the relevant authorities.

Serious adverse events (SAEs) will be reported within 24 hours of the investigator becoming aware, in accordance with Article 41 of CTR 536/2014.

- Suspected Unexpected Serious Adverse Reactions (SUSARs) will be reported to all investigators and Läkemedelsverket (LVM).

- All AEs and SAEs will be collected from Dosing 1 until final follow-up at 12 months.

9.4 Follow-up of Adverse Events

Follow-up visits will be scheduled for all participants experiencing AEs to ensure resolution and ongoing safety.

- Participants with unresolved AEs at the end of the trial will be monitored until resolution or stabilization.
- For SAEs, additional follow-ups will be scheduled at least every two weeks until resolution. The frequency can be changed by the Safety Review Committee or Principal Investigator.

9.5 Safety Review Committee

A Safety Review Committee will oversee trial safety and make recommendations on trial continuation. The committee will receive notification on all SAEs and SUARs by the research team within 24 hours.

The committee will review safety data quarterly and after every interim analysis (after every 10 psilocybin dosing sessions). They may recommend protocol modifications, halting recruitment, or terminating the trial based on emerging risks.

The committee will include at least two clinical and senior psychiatrists, a clinical pharmacologist, and a statistician, not affiliated with the research team, but not from an independent organisation.

No formal Data Monitoring Committee will be used in this trial.

9.6 Annual Safety Report (ASR)

An annual safety report will be submitted to regulatory authorities by the study coordinator in collaboration with the principal investigator.

The report will include a summary of all AEs, SAEs, SUSARs, and risk-benefit analyses to date. Specific trends in adverse events by treatment arm will also be evaluated.

9.7 Procedures in Case of Emergencies and Overdose

- Emergency protocols are in place, including immediate medical care and monitoring.

- In case of an overdose, the participant will be transferred to an emergency facility. Emergency kits, including benzodiazepines for anxiety or seizures, will be available during all dosing sessions.

9.8 Pregnancy Management

Recommendations related to contraception and pregnancy testing in clinical trials based on version 1.2 available at https://www.hma.eu/about-hma/working-groups/clinical-trials-coordination-group.html.

Woman of childbearing potential (WOCBP) is defined as any biologically female person post-menarche until menopause, unless surgically sterilized.

9.8.1 Prevention of Pregnancy During the Study

- Participants of childbearing potential must agree to use a highly effective contraceptive method throughout their participation in the study and for at least two months following their final psilocybin administration session. Highly effective contraceptive methods: combined (estrogen and progestogen containing) contraception associated with inhibition of ovulation, oral intravaginal transdermal hormonal, progestogen-only hormonal contraception associated with inhibition of ovulation, oral injectable implantable, intrauterine device (IUD), intrauterine hormone-releasing system, bilateral tubal occlusion, vasectomised partner, sexual abstinence.

9.8.2 Pregnancy Testing

- All participants of childbearing potential will undergo a urine pregnancy test during screening.
- A urine pregnancy test will be conducted at baseline (prior to the first psilocybin administration) and before the second subsequent psilocybin dosing session.
- Additional pregnancy tests may be performed if clinically indicated during follow-up visits or as deemed necessary by the investigator.

9.8.3 Exclusion of Pregnant Participants

- Participants who are pregnant or nursing at screening or who plan to become pregnant during the study period will be excluded.
- Participants must confirm their commitment to avoiding pregnancy during the study as part of the informed consent process.

9.8.4 Immediate Action Upon Pregnancy Detection

- If a participant becomes pregnant during the study:
  - Psilocybin administration will be discontinued immediately.
  - Appropriate obstetric care referrals will be provided, and the participants will receive follow-up to ensure their well-being.

Rationale for Precautions

- Psilocybin’s impact on reproductive health and fetal development is unknown due to the lack of reproductive toxicology studies and therefore will be considered to belong to the risk category of “demonstrated/suspected teratogenicity”, in which the participants must use contraception considered to be highly effective as listed above. These measures aim to mitigate any potential risks and prioritize participant safety.

10. Statistics

10.1 Analysis Population

- Both the Intention-to-treat (ITT) and per-protocol populations will be analysed.

ITT analysis will include all participants who are randomized, regardless of protocol adherence, to ensure generalizability.

Per-protocol analysis will focus on participants who completed the study as planned, ensuring the assessment of efficacy under ideal conditions.

The trial aims to dose at least 20 participants in the psilocybin treatment arm. This number refers to participants who receive at least one 25 mg psilocybin session, not only those who are randomized.

Analyses of safety and tolerability will primarily focus on this dosed cohort.

Recruitment will continue until a minimum of 20 participants in the intervention arm have received at least one dose, unless safety or feasibility concerns require early termination.

10.2 Statistical Analyses

This will be an open label, two-arm, active comparator (TAU; treatment as usual), , randomized controlled, between- and within-subjects, design in up to 40 completing patients meeting DSM-5 criteria for eating disorders. The patients will be randomly allocated to the psilocybin treatment-arm. The study analyses will enable comparison of efficacy between psilocybin-treatment and treatment as usual, as well as longitudinal within-patient efficacies.

- Primary Baseline Analyses:
  The primary analyses will involve descriptive statistics for demographic and baseline characteristics, ensuring comparability across groups, and control for follow-up measurements.
- Primary Endpoints analysis
- We will analyse differences in the number of participants and severity experiencing AE/SAE between the groups standardized forms for AE/SAE capturing: Event description, Start and end dates, Severity (e.g., mild, moderate, severe), Relatedness to intervention (assessed by safety review committee), Action taken.

The primary statistical methods will be:

- Descriptive Frequencies and Percentages.
- Comparing Proportions (Most Common for "Incidence") (Chi-squared test (or Fisher's Exact Test): Fisher's exact test is preferred for small cell counts (<5).
- Risk Ratio (RR) or Odds Ratio (OR) with Confidence Intervals: Report these measures of effect size to quantify the magnitude and precision of the difference between groups. An RR > 1 would indicate a higher risk in the intervention group.
- Comparing Severity and Relatedness is assessed with Mann-Whitney U test or Student t-test to compare severity distributions between groups.
- Secondary Endpoints:
  For secondary endpoints (e.g., changes in fMRI connectivity, BDNF, rating scales), group comparisons, including t-test, ANOVA, and Repeated Measures ANOVA will be utilized. When controlling for variables such as individual differences, ANCOVA or MANCOVA will be utilized. Principal component analysis (PCA) or independent component analysis (ICA) may be applied to identify patterns in fMRI data. Task-based fMRI data will be analysed using univariate models to investigate treatment effects in syndrome-related neural circuits (Celeghin et al., 2023; Bronleigh et al., 2022). Endpoints include longitudinal between- and within-person analyses.
- When dichotomous (binary) outcome variables: Binary outcomes (e.g., remission, response rates) will be analyzed using logistic regression models, adjusting for baseline characteristics such as age, baseline BMI, and symptom severity. The odds ratios and 95% confidence intervals will be reported.
- When continuous (dimensional) variables (e.g., BMI, BDNF levels, cognitive flexibility) will be analyzed using linear mixed-effects models, and regression models of various types.
- Other: Exploratory Subgroup Analyses:
  Exploratory subgroup analyses will assess treatment effects across different strata (e.g., age groups, baseline severity) using interaction terms in regression models or stratified analyses to explore heterogeneity in treatment responses.
- Other: Sensitivity Analyses:
  Sensitivity analyses will address missing data using methods such as multiple imputation or maximum likelihood estimation. These methods ensure robustness of the findings by accounting for the potential impact of missing data on primary and secondary outcomes.

**Concomitant antidepressant treatment (yes/no; class; dose stability)** will be recorded and may be included as a covariate in exploratory analyses. Sensitivity analyses or subgroup analyses may be performed to explore potential moderating effects on clinical outcomes.

10.3 Adjustment of Significance and Confidence Interval

A Bonferroni correction or false discovery rate (FDR) adjustment will be applied for multiple comparisons to control Type I error.

Results will be presented with 95% confidence intervals, and significance will be set at a two-tailed p-value of <0.05 after adjustments.

10.4 Sample Size Calculations

No formal power calculation has been conducted due to the pilot nature of the study. However, 40 participants (20 per group) were chosen based on feasibility and alignment with recommendations for exploratory trials in emerging therapies​.

Post-hoc power analysis will assess the adequacy of the sample size in detecting clinically meaningful changes in primary outcomes.

10.5 Interim Analysis

Following two administration sessions of 25mg psilocybin, a panel of three senior psychiatrists, who are not part of the research team, will conduct an evaluation of the safety data and adherence to the protocol. This analysis will be repeated after a total of 20 psilocybin administrations have been completed. After 25 patients over 18 have been through dosing sessions, patients 16-17 will be recruited.

11. Quality Control and Quality Assurance

11.1 Quality Assurance and Sponsor Oversight

Continuous oversight by the sponsor, including audits and video monitoring of all psilocybin dosing sessions.

11.2 Monitoring

The study will be subject to ongoing monitoring to ensure protocol adherence, data quality, regulatory compliance, and participant safety. Monitoring will be conducted in accordance with Good Clinical Practice (GCP), the EU Clinical Trials Regulation (CTR), and Swedish regulatory requirements.

**Monitoring Oversight and Responsibilities**

- The sponsor is responsible for ensuring proper monitoring, which will be conducted by appointed and qualified, independent monitors in collaboration with Kliniska Studier, Forum Söder, Lund, who are not directly involved in trial execution.
- The monitoring process will be outlined by the monitors in a predefined Monitoring Plan, specifying the scope, frequency, and methodology of monitoring activities, including on-site visits, centralized monitoring, and remote data reviews.
- Monitoring will only commence once all trial prerequisites are in place, including:
  - Regulatory approvals from CTIS, Läkemedelsverket (LVM), and the Swedish Ethical Review Authority.
  - Complete site initiation and staff training in GCP and study procedures.
  - Implementation of a site-specific risk assessment plan.

**Monitoring Procedures**

Monitoring will include the following key elements:

- **Qualified Monitors Will Oversee the Study to Verify:**
  - That the rights, well-being, and safety of participants are protected.
  - That all protocol procedures are followed, including adherence to inclusion/exclusion criteria, informed consent, and investigational treatment administration.
  - That all data collected in the electronic Case Report Form (eCRF) is accurate, complete, and verifiable from source documents.
  - That adverse event (AE) reporting follows regulatory guidelines and timelines.
  - Serious adverse events (SAEs) will be reported within 24 hours of the investigator becoming aware, in accordance with Article 41 of CTR 536/2014.
  - That all study personnel maintain GCP compliance and are adequately trained.
- All AEs and SAEs will be collected from first dosing until final follow-up at 12 months.

**Monitoring Methods and Risk-Based Approach**

- - The study will follow a risk-based monitoring strategy, prioritizing sites and procedures with higher potential risks.
  - Monitoring will include:
    - On-site monitoring: Regular site visits to verify compliance with the protocol, inspect records, and resolve queries.
    - Centralized monitoring: Remote review of data consistency, trends, and site performance to detect potential protocol deviations or data anomalies.
    - Remote data monitoring: Ongoing electronic data review to identify missing data, inconsistencies, and protocol violations.
- **Deviation Reporting and Corrective Actions**
  - Any deviations from the protocol or regulatory requirements identified during monitoring will be documented, reported, and, if necessary, corrected through Corrective and Preventive Actions (CAPA).
  - Significant findings may require a site retraining, amendment implementation, or regulatory reporting to ensure compliance.

11.3 Source Data

- All source data will be documented in the REDCap documentation system.

11.4 Deviations, Serious Breaches, and Other Reporting Obligations

- Documented and reported according to regulatory requirements.

11.5 Audits and Inspections

This clinical trial will be subject to external audits and regulatory inspections to ensure compliance with Good Clinical Practice (GCP), the EU Clinical Trials Regulation (CTR), and Swedish national regulations.

Regulatory Inspections by Läkemedelsverket (LVM)

- The study will be open to inspections by **Läkemedelsverket (LVM)** at any stage of the trial, including during trial conduct, study closeout, and post-study archiving.

**External Audits**

- The study may be subject to **external audits** conducted by independent auditors assigned by the sponsor or regulatory authorities.

**Investigator Responsibilities During Inspections and Audits**

- Investigators and site personnel must ensure that all trial-related records are complete, accurate, and readily available for review.
- Any requests from regulatory authorities or auditors must be addressed promptly and transparently.
- Any findings requiring corrective action must be resolved in compliance with GCP and sponsor directive

12. Ethics

12.1 Compliance to the Protocol, ICH-GCP, and Regulations

This study will be conducted in full compliance with the EU Clinical Trials Regulation (CTR), ICH-GCP, the Declaration of Helsinki, and relevant Swedish laws and regulations governing clinical research.

12.2 Ethical Review of the Trial

**Submission and Approval Process**

- The study will first be submitted to the Clinical Trials Information System (CTIS), the study will then be submitted to the Swedish Ethical Review Authority (Etikprövningsmyndigheten) for national-level ethical approval.
- Approval from Läkemedelsverket is also required.
- The study will not commence until all necessary approvals have been obtained from both EU (CTIS) and Swedish regulatory authorities.

**Protocol Amendments**

- Any substantial amendments to the study protocol, including changes in study design, endpoints, participant eligibility criteria, or safety procedures, will be submitted to the Swedish Ethical Review Authority and CTIS for approval before implementation.
- Minor amendments that do not affect participant safety, study integrity, or regulatory compliance will be documented and communicated in accordance with GCP and regulatory guidelines.

12.3 Procedure for Obtaining Informed Consent

- Informed consent will be obtained from all participants, including both parents or other caregiver when patient is between 16-17.

- Participants will receive detailed written and verbal information about the trial's purpose, procedures, potential risks, and benefits. They will have the opportunity to ask questions and consult with family members or advisors before consenting. Care will be taken to ensure that minors understand the study in an age-appropriate manner.

- A cooling-off period of two weeks will be provided between the informed consent signing and baseline to further allow participants to make an informed decision before active treatment

12.4 Data Protection

- Data will be pseudonymized and handled according to GDPR.

Only study personnel with relevant clearance will have access to identifiable data. Pseudonymized data will be stored securely on encrypted servers.

- A data management plan (DMP) will outline the procedures for data collection, storage, sharing, and destruction, ensuring compliance with GDPR and ethical standards.

- Informed consent will include explicit information about data handling, potential use in future research, and participants’ right to withdraw consent for data use at any time.

12.5 Insurances

- Insurance for participants provided by the sponsor.

13. Substantial Changes to the Trial

- Substantial modifications will be reported to authorities via CTIS.

14. Collection, Handling, and Archiving of Data

14.1 Case Report Form (CRF)

Data for this study will be collected and managed using an electronic Case Report Form (eCRF) developed and maintained by Kliniska Studier Forum Söder. The eCRF system complies with Good Clinical Practice (GCP) and General Data Protection Regulation (GDPR) to ensure the confidentiality, integrity, and security of participant data.

**Data Collection and Management**

- The eCRF will be securely hosted ensuring that only authorized personnel have access to specific data relevant to their responsibilities.
- Study investigators and designated study personnel will enter data into the eCRF and all data entries will be timestamped with a digital audit trail to track modifications and ensure data traceability.
- Source data verification (SDV) will be performed as required, and any discrepancies identified during monitoring will be documented and resolved in accordance with the study's data management plan.

**Compliance with GDPR and Data Protection Measures**

To protect participants' privacy, all personal data will be **pseudonymized** or anonymized where possible, with identifiable information stored separately from research data. Data collection and processing will adhere to GDPR principles.

**Data Access and Storage**

- The sponsor, investigators, and designated study personnel will have access to the de-identified research data in the eCRF system based on role-specific permissions.
- Monitors, auditors, and regulatory authorities (e.g., the European Medicines Agency (EMA) and national regulatory agencies) will have controlled access to the data for oversight purposes.
- Data security measures include encrypted storage, secure login authentication, and routine system backups to prevent data loss.

**Investigator Site File (Prövarpärm)**

- A physical or electronic Investigator Site File (ISF), also known as the prövarpärm, will be maintained at each study site, containing all essential documents, including study logs, protocol amendments, informed consent forms, and monitoring reports.
- A Data Management Plan (DMP) outlining data handling procedures, security measures, and retention policies will also be included.

**Data Retention and Security Measures**

- All study data will be retained for a minimum of 25 years post-study completion, as required by clinical trial regulations.
- Secure long-term storage will be ensured by Kliniska Studier Forum Söder in compliance with data archiving policies, and access will be restricted to authorized personnel only.
- Upon study closure, the database will be locked to prevent further modifications, and final datasets will be archived in compliance with EMA and national regulatory requirements.

15. Notification of Trial Completion, Reporting, and Publication

The study team aims to present the findings at psychiatry and psychedelic science conferences. Publish in high-impact journals focusing on psychedelics, psychiatry, neurology, and pharmacology. Press releases to major healthcare news outlets and the public. Host webinars, lectures and participate in forums for mental health professionals and researchers in Sweden. Share findings with collaborating academic institutions for further research and teaching. Release follow-up studies and long-term effect analyses.

CTIS will be reported in CTIS within one year after completion of study (six months follow-up of active arm and controls, not including the switch).

16. References

Arcelus, J., Mitchell, A., Wales, J., & Nielsen, S. (2011). Mortality rates in patients with anorexia nervosa and other eating disorders. *Archives of General Psychiatry, 68*(7), 724. https://doi.org/10.1001/archgenpsychiatry.2011.74

Barrett, F. S., Doss, M. K., Sepeda, N. D., Pekar, J. J., & Griffiths, R. R. (2020). Emotions and brain function are altered up to one month after a single high dose of psilocybin. *Scientific Reports, 10*(1), 2214. <https://doi.org/10.1038/s41598-020-59282-y>

Bates, M. L. S., & Trujillo, K. A. (2021). Use and abuse of dissociative and psychedelic drugs in adolescence. *Pharmacology Biochemistry and Behavior, 203*, 173129. https://doi.org/10.1016/j.pbb.2021.173129

Bogenschutz, M. P., Ross, S., Bhatt, S., et al. (2022). Percentage of heavy drinking days following psilocybin-assisted psychotherapy vs placebo in the treatment of adult patients with alcohol use disorder: A randomized clinical trial. *JAMA Psychiatry, 79*(10), 953–962. https://doi.org/10.1001/jamapsychiatry.2022.2096

Bronleigh, M., Baumann, O., & Stapleton, P. (2022). Neural correlates associated with processing food stimuli in anorexia nervosa and bulimia nervosa: An activation likelihood estimation meta-analysis of fMRI studies. *Eating and Weight Disorders, 27*(7), 2309–2320. <https://doi.org/10.1007/s40519-022-01390-x>

Calder, A., Mock, S., Friedli, N., Pasi, P., & Hasler, G. (2023). Psychedelics in the treatment of eating disorders: Rationale and potential mechanisms. *European Neuropsychopharmacology, 75*, 1–14. <https://doi.org/10.1016/j.euroneuro.2023.05.008>

Carbonaro, T. M., Bradstreet, M. P., Barrett, F. S., MacLean, K. A., Jesse, R., Johnson, M. W., & Griffiths, R. R. (2016). Survey study of challenging experiences after ingesting psilocybin mushrooms: Acute and enduring positive and negative consequences. *Journal of Psychopharmacology, 30*(12), 1268–1278. https://doi.org/10.1177/0269881116662634

Carhart-Harris, R. L., Erritzoe, D., Williams, T., Stone, J. M., Reed, L. J., Colasanti, A., Tyacke, R. J., Leech, R., Malizia, A. L., Murphy, K., Hobden, P., Evans, J., Feilding, A., Wise, R. G., & Nutt, D. J. (2012). Neural correlates of the psychedelic state as determined by fMRI studies with psilocybin. *Proceedings of the National Academy of Sciences, 109*(6), 2138–2143. <https://doi.org/10.1073/pnas.1119598109>

Carhart-Harris, R. L., & Nutt, D. J. (2013). Experienced drug users assess the relative harms and benefits of drugs: A web-based survey. *Journal of Psychoactive Drugs, 45*(4), 322–328. <https://doi.org/10.1080/02791072.2013.825034>

Carhart-Harris, R. L., Bolstridge, M., Rucker, J., Day, C. M. J., Erritzoe, D., Kaelen, M., Bloomfield, M., Rickard, J. A., Forbes, B., Feilding, A., Taylor, D., Pilling, S., Curran, V. H., & Nutt, D. J. (2016a). Psilocybin with psychological support for treatment-resistant depression: An open-label feasibility study. *The Lancet Psychiatry, 3*(7), 619–627. <https://doi.org/10.1016/S2215-0366(16)30065-7>

Carhart-Harris, R. L., Kaelen, M., Bolstridge, M., Williams, T. M., Williams, L. T., Underwood, R., Feilding, A., & Nutt, D. J. (2016b). The paradoxical psychological effects of lysergic acid diethylamide (LSD). *Psychological Medicine, 46*(7), 1379–1390. <https://doi.org/10.1017/S0033291715002901>

Carhart-Harris, R. L., & Goodwin, G. M. (2017). The therapeutic potential of psychedelic drugs: Past, present, and future. *Neuropsychopharmacology, 42*(11), 2105–2113. https://doi.org/10.1038/npp.2017.84

Carhart-Harris, R., Giribaldi, B., Watts, R., Baker-Jones, M., Murphy-Beiner, A., Murphy, R., Martell, J., Blemings, A., Erritzoe, D., & Nutt, D. J. (2021). Trial of psilocybin versus escitalopram for depression. *New England Journal of Medicine, 384*(15), 1402–1411. <https://doi.org/10.1056/NEJMoa2032994>

Celeghin, A., Palermo, S., Giampaolo, R., Di Fini, G., Gandino, G., & Civilotti, C. (2023). Brain Correlates of Eating Disorders in Response to Food Visual Stimuli: A Systematic Narrative Review of FMRI Studies. Brain sciences, 13(3), 465. <https://doi.org/10.3390/brainsci13030465>

Cohen-Cory, S., Kidane, A. H., Shirkey, N. J., & Marshak, S. (2010). Brain-derived neurotrophic factor and the development of structural neuronal connectivity. *Developmental Neurobiology, 70*(5), 271–288. <https://doi.org/10.1002/dneu.20774>

Davis, A. K., Barrett, F. S., May, D. G., Cosimano, M. P., Sepeda, N. D., Johnson, M. W., Finan, P. H., & Griffiths, R. R. (2021). Effects of psilocybin-assisted therapy on major depressive disorder. *JAMA Psychiatry, 78*(5), 481–489. <https://doi.org/10.1001/jamapsychiatry.2020.3285>

Daws, R. E., Timmermann, C., Giribaldi, B., Sexton, J. D., Wall, M. B., Erritzoe, D., Roseman, L., Nutt, D., & Carhart-Harris, R. (2022). Increased global integration in the brain after psilocybin therapy for depression. *Nature Medicine, 28*(4), 844–851. <https://doi.org/10.1038/s41591-022-01744-z>

DeJong, H., Oldershaw, A., Sternheim, L., Samarawickrema, N., Kenyon, M. D., Broadbent, H., Lavender, A., Startup, H., Treasure, J., & Schmidt, U. (2013). Quality of life in anorexia nervosa, bulimia nervosa and eating disorder not-otherwise-specified. *Journal of Eating Disorders, 1*(1), 43. https://doi.org/10.1186/2050-2974-1-43

Foldi, C. J., Liknaitzky, P., Williams, M., & Oldfield, B. J. (2020). Rethinking therapeutic strategies for anorexia nervosa: Insights from psychedelic medicine and animal models. *Frontiers in Neuroscience, 14*, 43. <https://doi.org/10.3389/fnins.2020.00043>

Fuglset, T. S., Landrø, N. I., Reas, D. L., & Rø, Ø. (2016). Functional brain alterations in anorexia nervosa: A scoping review. *Journal of Eating Disorders, 4*, 32. <https://doi.org/10.1186/s40337-016-0118-y>

Galvão-Coelho, N. L., Marx, W., Gonzalez, M., Sinclair, J., de Manincor, M., Perkins, D., & Sarris, J. (2021). Classic serotonergic psychedelics for mood and depressive symptoms: A meta-analysis of mood disorder patients and healthy participants. *Psychopharmacology, 238*(2), 341–354. <https://doi.org/10.1007/s00213-020-05719-1>

Garcia-Romeu A, Griffiths RR, Johnson MW. Psilocybin-occasioned mystical experiences in the treatment of tobacco addiction. Curr Drug Abuse Rev. 2014;7(3):157-64. doi: 10.2174/1874473708666150107121331. PMID: 25563443; PMCID: PMC4342293.

Goldberg, S. B., Pace, B. T., Nicholas, C. R., Raison, C. L., & Hutson, P. R. (2020). The experimental effects of psilocybin on symptoms of anxiety and depression: A meta-analysis. *Psychiatry Research, 284*, 112749. https://doi.org/10.1016/j.psychres.2020.112749

Griffiths, R. R., Richards, W. A., Johnson, M. W., McCann, U., & Jesse, R. (2008). Mystical-type experiences occasioned by psilocybin mediate the attribution of personal meaning and spiritual significance 14 months later. *Journal of Psychopharmacology, 22*(6), 621–632. https://doi.org/10.1177/0269881108094300

Griffiths, R. R., Richards, W. A., McCann, U., & Jesse, R. (2006). Psilocybin can occasion mystical-type experiences having substantial and sustained personal meaning and spiritual significance. *Psychopharmacology, 187*(3), 268–283. https://doi.org/10.1007/s00213-006-0457-5

Grob, C. S., Danforth, A. L., Chopra, G. S., Hagerty, M., McKay, C. R., Halberstadt, A. L., & Greer, G. R. (2011). Pilot study of psilocybin treatment for anxiety in patients with advanced-stage cancer. *Archives of General Psychiatry, 68*(1), 71–78. <https://doi.org/10.1001/archgenpsychiatry.2010.116>

**Gukasyan N, Davis AK, Barrett FS, Cosimano MP, Sepeda ND, Johnson MW, Griffiths RR. Efficacy and safety of psilocybin-assisted treatment for major depressive disorder: Prospective 12-month follow-up. J Psychopharmacol. 2022 Feb;36(2):151-158. doi: 10.1177/02698811211073759. PMID: 35166158; PMCID: PMC8864328.**Halmi, K. A. (2013). Perplexities of treatment resistance in eating disorders. *BMC Psychiatry, 13*(1), 292. <https://doi.org/10.1186/1471-244X-13-292>

Halmi, K., Goldberg, S., Casper, R., Eckert, E., & Davis, J. (1979). Pretreatment predictors of outcome in anorexia nervosa. *British Journal of Psychiatry, 134*(1), 71–78. <https://doi.org/10.1192/bjp.134.1.71>

Hendricks, P. S., Thorne, C. B., Clark, C. B., Coombs, D. W., & Johnson, M. W. (2015). Classic psychedelic use is associated with reduced psychological distress and suicidality in the United States adult population. *Journal of Psychopharmacology, 29*(3), 280–288. <https://doi.org/10.1177/0269881114565653>

Hoek, H. (2006). Incidence, prevalence and mortality of anorexia nervosa and other eating disorders. *Current Opinion in Psychiatry, 19*(4), 389–394. https://doi.org/10.1097/01.yco.0000228759.95237.78

Johnson, M., Richards, W., & Griffiths, R. (2008). Human hallucinogen research: Guidelines for safety. *Journal of Psychopharmacology, 22*(6), 603–620. https://doi.org/10.1177/0269881108093587

Kaye, W. H., Fudge, J. L., & Paulus, M. (2009). New insights into symptoms and neurocircuit function of anorexia nervosa. *Nature Reviews Neuroscience, 10*(8), 573–584. <https://doi.org/10.1038/nrn2682>

Khalsa, S. S., Portnoff, L. C., McCurdy-McKinnon, D., & Feusner, J. D. (2017). What happens after treatment? A systematic review of relapse, remission, and recovery in anorexia nervosa. *Journal of Eating Disorders, 5*, 20. <https://doi.org/10.1186/s40337-017-0145-3>

Knutson, B., Westdorp, A., Kaiser, E., & Hommer, D. (2000). FMRI visualization of brain activity during a monetary incentive delay task. NeuroImage, 12(1), 20–27. <https://doi.org/10.1006/nimg.2000.0593>

Kurtom, M., Henning, A., & Espiridion, E. D. (2019). Hallucinogen-persisting perception disorder in a 21-year-old man. *Cureus, 11*(2), e4077. <https://doi.org/10.7759/cureus.4077>

Lafrance, A., Loizaga-Velder, A., Fletcher, J., Renelli, M., Files, N., & Tupper, K. W. (2017). Nourishing the spirit: Exploratory research on ayahuasca experiences along the continuum of recovery from eating disorders. *Journal of Psychoactive Drugs, 49*(5), 427–435. <https://doi.org/10.1080/02791072.2017.1361559>

Lafrance, A., Strahan, E., Bird, B. M., St. Pierre, M., & Walsh, Z. (2021). Classic psychedelic use and mechanisms of mental health: Exploring the mediating roles of spirituality and emotion processing on symptoms of anxiety, depressed mood, and disordered eating in a community sample. *Journal of Humanistic Psychology*. <https://doi.org/10.1177/00221678211048099>

Lebedev, A. V., Kaelen, M., Lövdén, M., Nilsson, J., Feilding, A., Nutt, D. J., & Carhart-Harris, R. L. (2016). LSD-induced entropic brain activity predicts subsequent personality change. *Human Brain Mapping, 37*(9), 3203–3213. <https://doi.org/10.1002/hbm.23234>

Liu, P., Ioannidis, J. P. A., Ross, J. S., Dhruva, S. S., Luxkaranayagam, A. T., Vasiliou, V., & Wallach, J. D. (2019). Age-treatment subgroup analyses in Cochrane intervention reviews: A meta-epidemiological study. *BMC Medicine, 17*(1), 1–9. <https://doi.org/10.1186/s12916-019-1420-8>

Lohof, A. M., Ip, N. Y., & Poo, M. M. (1993). Potentiation of developing neuromuscular synapses by the neurotrophins NT-3 and BDNF. *Nature, 363*(6427), 350–353. <https://doi.org/10.1038/363350a0>

Ly, C., Greb, A. C., Cameron, L. P., Wong, J. M., Barragan, E. V., Wilson, P. C., Burbach, K. F., Soltanzadeh Zarandi, S., Sood, A., Paddy, M. R., Duim, W. C., Dennis, M. Y., McAllister, A. K., Ori-McKenney, K. M., Gray, J. A., & Olson, D. E. (2018). Psychedelics Promote Structural and Functional Neural Plasticity. *Cell reports, 23*(11), 3170–3182. <https://doi.org/10.1016/j.celrep.2018.05.022>

MacCallum CA, Lo LA, Pistawka CA, Deol JK. Therapeutic use of psilocybin: Practical considerations for dosing and administration. Front Psychiatry. 2022 Dec 1;13:1040217. doi: 10.3389/fpsyt.2022.1040217. PMID: 36532184; PMCID: PMC9751063.

MacLean, Katherine. A., Johnson, Matthew. W., & Griffiths, Roland. R. (2013). Mystical Experiences Occasioned by the Hallucinogen Psilocybin Lead to Increases in the Personality Domain of Openness. *Journal of Psychopharmacology, 25*(11), 1453–1461. <https://doi.org/10.1177/0269881111420188>

Mason, N. L., Kuypers, K. P. C., Müller, F., Reckweg, J., Tse, D. H. Y., Toennes, S. W., Hutten, N. R. P. W., Jansen, J. F. A., Stiers, P., Feilding, A., & Ramaekers, J. G. (2020). Me, myself, bye: Regional alterations in glutamate and the experience of ego dissolution with psilocybin. *Neuropsychopharmacology, 45*(12), 2003–2011. https://doi.org/10.1038/s41386-020-0718-8

Mertens, L. J., & Preller, K. H. (2021). Classical psychedelics as therapeutics in psychiatry – Current clinical evidence and potential therapeutic mechanisms in substance use and mood disorders. *Pharmacopsychiatry*. <https://doi.org/10.1055/a-1341-1907>

Moliner, R., Girych, M., Brunello, C. A., Kovaleva, V., Biojone, C., Enkavi, G., Antenucci, L., Kot, E. F., Goncharuk, S. A., Kaurinkoski, K., Kuutti, M., Fred, S. M., Elsilä, L. V., Sakson, S., Cannarozzo, C., Diniz, C. R. A. F., Seiffert, N., Rubiolo, A., Haapaniemi, H., Meshi, E., & Castrén, E. (2023). Psychedelics promote plasticity by directly binding to BDNF receptor TrkB. *Nature Neuroscience, 26*(6), 1032–1041. <https://doi.org/10.1038/s41593-023-01316-5>

Moreno, F. A., Wiegand, C. B., Taitano, E. K., & Delgado, P. L. (2006). Safety, tolerability, and efficacy of psilocybin in 9 patients with obsessive-compulsive disorder. *The Journal of Clinical Psychiatry, 67*(11), 1735–1740. https://doi.org/10.4088/jcp.v67n1110

Nakazato, M., Hashimoto, K., Shimizu, E., Kumakiri, C., Koizumi, H., Okamura, N., Mitsumori, M., Komatsu, N., & Iyo, M. (2003). Decreased levels of serum brain-derived neurotrophic factor in female patients with eating disorders. *Biological Psychiatry, 54*(4), 485–490. <https://doi.org/10.1016/s0006-3223(02)01746-8>

Nour, M. M., Evans, L., Nutt, D., & Carhart-Harris, R. L. (2016). Ego-dissolution and psychedelics: Validation of the Ego-Dissolution Inventory (EDI). *Frontiers in Human Neuroscience, 10*. https://doi.org/10.3389/fnhum.2016.00269

Peck, S. K., Shao, S., Gruen, T., Yang, K., Babakanian, A., Trim, J., Finn, D. M., & Kaye, W. H. (2023). Psilocybin therapy for females with anorexia nervosa: A phase 1, open-label feasibility study. *Nature Medicine, 29*(8), 1947–1953. <https://doi.org/10.1038/s41591-023-02455-9>

Puckett, L., Grayeb, D., Khatri, V., Cass, K., & Mehler, P. (2021). A comprehensive review of complications and new findings associated with anorexia nervosa. *Journal of Clinical Medicine, 10*(12), 2555. <https://doi.org/10.3390/jcm10122555>

Rajwani, K. (2022). Should adolescents be included in emerging psychedelic research? *Canadian Journal of Bioethics / Revue Canadienne de Bioéthique, 5*(2), 36–43. <https://doi.org/10.7202/1089784ar>

Renelli, M., Fletcher, J., Tupper, K. W., Files, N., Loizaga-Velder, A., & Lafrance, A. (2018). An exploratory study of experiences with conventional eating disorder treatment and ceremonial ayahuasca for the healing of eating disorders. *Eating and Weight Disorders - EWD 25*(2), 437–444. <https://doi.org/10.1007/s40519-018-0619-6>

Siegel, J. S., Subramanian, S., Perry, D., Kay, B., Gordon, E., Laumann, T., Reneau, R., Gratton, C., Horan, C., Metcalf, N., Chacko, R., Schweiger, J., Wong, D., Bender, D., Padawer-Curry, J., Raison, C., Raichle, M., Lenze, E. J., Snyder, A. Z., … Nicol, G. (2023). Psilocybin desynchronizes brain networks. *medRxiv: The Preprint Server for Health Sciences.* <https://doi.org/10.1101/2023.08.22.23294131>

Steinhausen, H. (2002). The outcome of anorexia nervosa in the 20th century. *American Journal of Psychiatry, 159*(8), 1284–1293. <https://doi.org/10.1176/appi.ajp.159.8.1284>

Steinman, J., & Shibli-Rahhal, A. (2019). Anorexia nervosa and osteoporosis: Pathophysiology and treatment. *Journal of Bone Metabolism, 26*(3), 133–143. <https://doi.org/10.11005/jbm.2019.26.3.133>

Steward, T., Menchon, J. M., Jiménez-Murcia, S., Soriano-Mas, C., & Fernandez-Aranda, F. (2018). Neural network alterations across eating disorders: A narrative review of fMRI studies. *Current Neuropharmacology, 16*(8), 1150–1163. <https://doi.org/10.2174/1570159X15666171017111532>

Studerus, E., Kometer, M., Hasler, F., & Vollenweider, F. X. (2010). Acute, subacute and long-term subjective effects of psilocybin in healthy humans: A pooled analysis of experimental studies. *Journal of Psychopharmacology, 25*(11), 1434–1452. <https://doi.org/10.1177/0269881110382466>

Swanson, L. R. (2018). Unifying theories of psychedelic drug effects. *Frontiers in Pharmacology, 9.* <https://doi.org/10.3389/fphar.2018.00172>

Wall, M. B., Harding, R., Zafar, R., Rabiner, E. A., Nutt, D. J., & Erritzoe, D. (2023). Neuroimaging in psychedelic drug development: past, present, and future. *Molecular psychiatry*, *28*(9), 3573–3580. <https://doi.org/10.1038/s41380-023-02271-0>

Ventorp, F., Lindahl, J., van Westen, D., Jensen, J., Björkstrand, J., & Lindqvist, D. (2022). Preliminary Evidence of Efficacy and Target Engagement of Pramipexole in Anhedonic Depression. Psychiatric research and clinical practice, 4(2), 42–47. <https://doi.org/10.1176/appi.prcp.20210042>

Westmoreland, [Patricia](https://www.clinicalkey.com/#!/search/Westmoreland%20Patricia/%7B%22type%22:%22author%22%7D)., Krantz, Mori. J., Mehler, Philip. S (2016).Medical Complications of Anorexia Nervosa and Bulimia. *American Journal of Medicine, 129* (1), 30-37. https://doi.org/[10.1016/j.amjmed.2015.06.031](https://doi.org/10.1016/j.amjmed.2015.06.031)

Wolff, M., Evens, R., Mertens, L. J., Koslowski, M., Betzler, F., Gründer, G., & Jungaberle, H. (2020). Learning to let go: A cognitive-behavioral model of how psychedelic therapy promotes acceptance. *Frontiers in Psychiatry, 11*. <https://doi.org/10.3389/fpsyt.2020.00005>

Zipfel, S., Seibel, M. J., Löwe, B., Beumont, P. J., Kasperk, C., & Herzog, W. (2001). Osteoporosis in eating disorders: A follow-up study of patients with anorexia and bulimia nervosa. *The Journal of Clinical Endocrinology & Metabolism, 86*(11), 5227–5233. <https://doi.org/10.1210/jcem.86.11.8050>

Becker, A. M., Humbert-Droz, M., Mueller, L., Jelusic, A., Tolev, A., Straumann, I., Avedisian, I., Erne, L., Thomann, J., Luethi, D., Grunblatt, E., Meyer Zu Schwabedissen, H., & Liechti, M. E. (2025). Acute Effects and Pharmacokinetics of LSD after Paroxetine or Placebo Pre-Administration in a Randomized, Double-Blind, Cross-Over Phase I Trial. *Clin Pharmacol Ther*. <https://doi.org/10.1002/cpt.3618>

Gukasyan, N., Griffiths, R. R., Yaden, D. B., Antoine, D. G., 2nd, & Nayak, S. M. (2023). Attenuation of psilocybin mushroom effects during and after SSRI/SNRI antidepressant use. *J Psychopharmacol*, *37*(7), 707-716. <https://doi.org/10.1177/02698811231179910>
